# Carotenoid accumulation affects redox status, starch metabolism, and flavonoid/anthocyanin accumulation in citrus

**DOI:** 10.1186/s12870-015-0426-4

**Published:** 2015-02-03

**Authors:** Hongbo Cao, Jiangbo Wang, Xintian Dong, Yan Han, Qiaoli Ma, Yuduan Ding, Fei Zhao, Jiancheng Zhang, Haijiang Chen, Qiang Xu, Juan Xu, Xiuxin Deng

**Affiliations:** Key Laboratory of Horticultural Plant Biology (Ministry of Education), Huazhong Agricultural University, 430070 Wuhan, Hubei China; College of Horticulture, Agricultural University of Hebei, 071001 Baoding, Hebei China; Present address: College of Plant Science, Tarim University, 843300 Alar, China; Present address: Shanxi Agricultural University, 030801 Taigu, Shanxi China

**Keywords:** Carotenogenesis, Citrus, Redox status, Starch, Chromoplast, Anthocyanin

## Abstract

**Background:**

Carotenoids are indispensable plant secondary metabolites that are involved in photosynthesis, antioxidation, and phytohormone biosynthesis. Carotenoids are likely involved in other biological functions that have yet to be discovered. In this study, we integrated genomic, biochemical, and cellular studies to gain deep insight into carotenoid-related biological processes in citrus calli overexpressing CrtB (phytoene synthase from *Pantoea agglomerans*). *Fortunella hindsii* Swingle (a citrus relative) and *Malus hupehensis* (a wild apple) calli were also utilized as supporting systems to investigate the effect of altered carotenoid accumulation on carotenoid-related biological processes.

**Results:**

Transcriptomic analysis provided deep insight into the carotenoid-related biological processes of redox status, starch metabolism, and flavonoid/anthocyanin accumulation. By applying biochemical and cytological analyses, we determined that the altered redox status was associated with variations in O_2_^-^ and H_2_O_2_ levels. We also ascertained a decline in starch accumulation in carotenoid-rich calli. Furthermore, via an extensive cellular investigation of the newly constructed *CrtB* overexpressing *Fortunella hindsii* Swingle, we demonstrated that starch level reducation occurred in parallel with significant carotenoid accumulation. Moreover, studying anthocyanin-rich *Malus hupehensis* calli showed a negative effect of carotenoids on anthocyanin accumulation.

**Conclusions:**

In citrus, altered carotenoid accumulation resulted in dramatic effects on metabolic processes involved in redox modification, starch degradation, and flavonoid/anthocyanin biosynthesis. These findings provided new perspectives to understand the biological importance of carotenogenesis and of the developmental processes associated with the nutritional and sensory qualities of agricultural products that accumulate carotenoids.

**Electronic supplementary material:**

The online version of this article (doi:10.1186/s12870-015-0426-4) contains supplementary material, which is available to authorized users.

## Background

Carotenoids, which first appeared in bacteria over three billion years ago, belong to a subfamily of isoprenoids that are commonly found in all organisms [1]. In nature, carotenoids originate from the condensation of geranylgeranyl pyrophosphate (GGPP), which is derived from the synthesis of isoprenoid precursors via the plastid-localized 2-C-methyl-D-erythritol 4-phosphate (MEP) pathway. In the crucial rate-controlling step, phytoene synthase (PSY) mediates the condensation of GGPP, forming the first carotenoid, phytoene [2,3]. Subsequently, different types of carotenoids are generated through various synthetic pathways including desaturation, isomerization, cyclization, hydroxylation, and other modifications [3]. Carotenoids and their derivatives play essential physiological and ecological roles. They are involved in the photosynthetic apparatus, photoprotective pigments, antioxidants, hormone precursors, and attractants for pollinators in plant growth, development, and reproduction [2]. Carotenoids are the precursors of phytohormones such as abscisic acid, strigolactones and the recently identified carlactone, which negatively regulates plant axillary outgrowth [4-6]. Ramel et al. [7] recently revealed that a carotenoid endoperoxide that originates from a reaction between β-carotene and reactive oxygen species (ROS) can serve as a stress signal that mediates gene responses to singlet oxygen in *Arabidopsis*. In addition, an epistatic influence on the expression of endogenous carotenogenic genes has been observed in carotenoid-engineered potato tubers, which suggests a feedback regulation of carotenoid metabolites [8,9]. It is reasonable to hypothesize that there are still more biological functions associated with carotenoids or their derivatives that are waiting to be uncovered.

Due to the health promoting function of carotenoids, research on their biosynthesis and accumulation in plants has been a predominant focus. However, knowledge of the effects of carotenoid metabolism on other plant processes is still relatively limited. In natural systems, such as in the fruits of citrus and tomato, carotenoid biosynthesis and accumulation often occur in parallel with the ripening process [10,11]. It is quite certain that the ripening process involves cellular activities related to metabolic networks and to organelle modification, which eventually determine product quality. In addition to playing a role in fruit quality, some metabolites, such as malate and anthocyanin, have newly discovered physiological functions associated with the regulation of fruit metabolism, development, and shelf life [12,13]. Fraser et al. [14] discovered the effects of enhanced carotenoid accumulation on isoprenoids, plastid development, and intermediary metabolism in tomato fruits. In investigations of carotenoid-accumulating citrus mutants, some biological processes associated with carbohydrate metabolism and oxidative stress were found to differ from the wild types [15,16]. Relatively little is known about how the carotenoid accumulation program might contribute to these unintended metabolic changes.

Chromoplast formation is one of the most important cellular changes during the ripening of carotenoid-rich plant tissues; it involves significant carotenoid sequestration and the use of other metabolic pathways, which are all essential for the nutritional and sensory quality of agricultural products [17]. Chromoplasts are generally derived from preexisting plastids such as amyloplasts or chloroplasts, and the chromoplast transition is often associated with tissue and organ development [18]. During chromoplast development, plastoglobules and carotenoid crystals form, starch breakdown occurs, starch granules and thylakoids disappear, and the metabolism of terpenoids and lipids is greatly enhanced [11,17,19,20]. To date, there is limited understanding of how the chromoplast developmental program is established [18], and the associative inner system including metabolic variation and structural remodeling still exhibits intricate behavior. In recent years, a considerable amount of research has shown that chromoplast biogenesis is mediated by crucial factors such as Orange (OR, a DnaJ cysteine-rich domain-containing protein) and CHRC (chromoplast-specific carotenoid-associated protein) [18,21]. Furthermore, in tobacco floral nectaries and carrot roots, the mutually exclusive relationship between carotenoid accumulation and starch granule development suggests that enhanced carotenogenesis serves as a developmental signal that directs the transition from amyloplasts to chromoplasts [19,22]. Additionally, modification of chromoplast morphology has been previously observed in carotenoid engineered plants, which suggests the possibility that cellular structures can adapt to facilitate the sequestration of newly formed carotenoids [23].

In addition to carotenoid biosynthesis, anthocyanin accumulation is an important biological event during the ripening of some fruits. Anthocyanins are plant phenolic secondary metabolites that are part of the phenylpropanoid pathway [24]. Like carotenoids, they are involved in a series of pivotal biological processes, such as antioxidative protection and the producion of attractants for reproduction, and they are also essential for the protection of human health [13]. Anthocyanins can co-exist with carotenoids in plant tissues and organs, but in some carotenoid-rich organs, there is relatively little anthocyanin accumulation. This phenomenon was observed in *Oncidium* Gower Ramsey flowers and in tomato fruits [25,26]. It is unknown if there is a negative correlation between carotenoid and anthocyanin, and it is a difficult question to answer. Carotenoids and anthocyanins are often involved in concurrent biosynthetic processes that accompany natural development, which makes it difficut to define a causal relationship. Similar observations have also been made for other hypothesized carotenoid-associated biological processes. Recently, genetic manipulation has been used successfully in many plants to modify carotenogenesis and other quality-associated components [27]. In these engineered plants, which have targeted metabolic pathway modifications, various unintended physiological, biochemical, and cellular changes have occurred [12-14]. Engineered systems appear to provide an effective approach for regulating the accumulation of a given metabolite, and they can facilitate the identification of associative biological relationships [12,13].

In our previous study, engineered cell models (ECMs) were established by activating the rate-controlling reaction by overexpressing the CrtB protein (phytoene synthase from *Erwinia herbicola*, now known as *Pantoea agglomerans*) in citrus embryogenic calli [28]. These ECMs exhibit diverse colors and accumulate significant levels of carotenoids. They are useful for understanding not only carotenoid biosynthesis, but also the potential biological processes associated with carotenoid accumulation. In the present study, we use Affymetrix microarrays, biochemistry, and cellular investigation of citrus calli to gain deeper insight into carotenoid-related biological processes, including redox status alternation, starch metabolism, and decreased flavonoid/anthocyanin accumulation. Engineering these pathways in *Fortunella hindsii* Swingle (a citrus relative) and *Malus hupehensis* (a wild apple) calli further validate these results.

## Results

### ECM transcriptional patterns

Engineered cell models (ECMs) generated by over-expressing 35S:: *CrtB* in citrus embryogenic calli show a striking accumulation of carotenoids [28]. However, relatively little is known about the other biological processes associated with engineered carotenoid accumulation. Thus, to further comprehend the cellular responses to enhanced carotenoid biosynthesis, we used three representative ECMs (M-33, from Marsh grapefruit; RB-4, from Star Ruby grapefruit; and SBT-6, from Sunburst mandarin) and their wild types in Affymetrix microarray analysis. Genes that were up- or down-regulated more than 2-fold (ECMs/WTs, *P* < 0.05) were identified, but a relaxed threshold of 1.5-fold (ECMs/WTs, *P* < 0.05) was used for genes in the M-33/WT microarray data (Additional file 1). This relaxed threshold was utilized because there was a relative lack of differentially expressed genes between M-33 and its wild type, most likely due to strong acclimation to carotenoid accumulation in the Marsh grapefruit genotype. To validate the microarray data, a quantitative real-time PCR (qRT-PCR) experiment was performed on the three representative ECMs and their wild types. A total of 10 genes were selected, and gene-specific primers were designed. Linear regression analysis showed an overall correlation coefficient of *R*^*2*^ = 0.6605 between the qRT-PCR and microarray data, which confirmed that the microarray data were reliable (Additional file 2).

**Figure 1:**
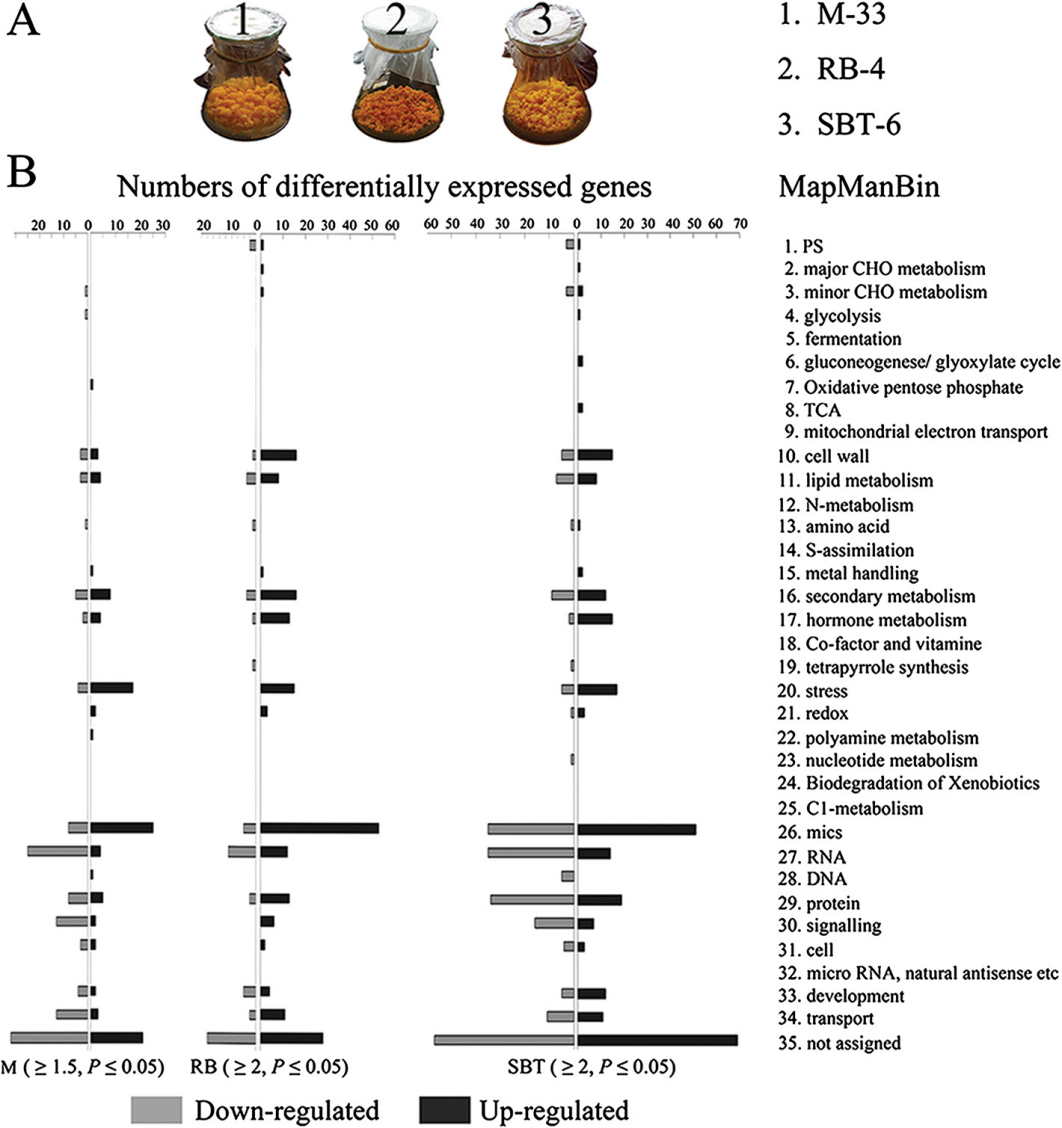
**The MapMan Bin of differentially expressed genes showed that three genotypes behaved similarly in their transcriptional patterns. (A)** Three representative ECMs used in the microarray analysis. M-33, RB-4, and SBT-6 were the representative ECMs of Marsh grapefruit, Star Ruby grapefruit, and Sunburst mandarin, respectively. **(B)** MapMan Bin of differentially expressed genes. Genes up/down-regulated more than 2-fold (ECMs/WTs, *P* < 0.05) are collected in RB and SBT, and more than 1.5-fold (ECMs/WTs, *P* < 0.05) are regarded as differentially expressed in M.

**Figure 2:**
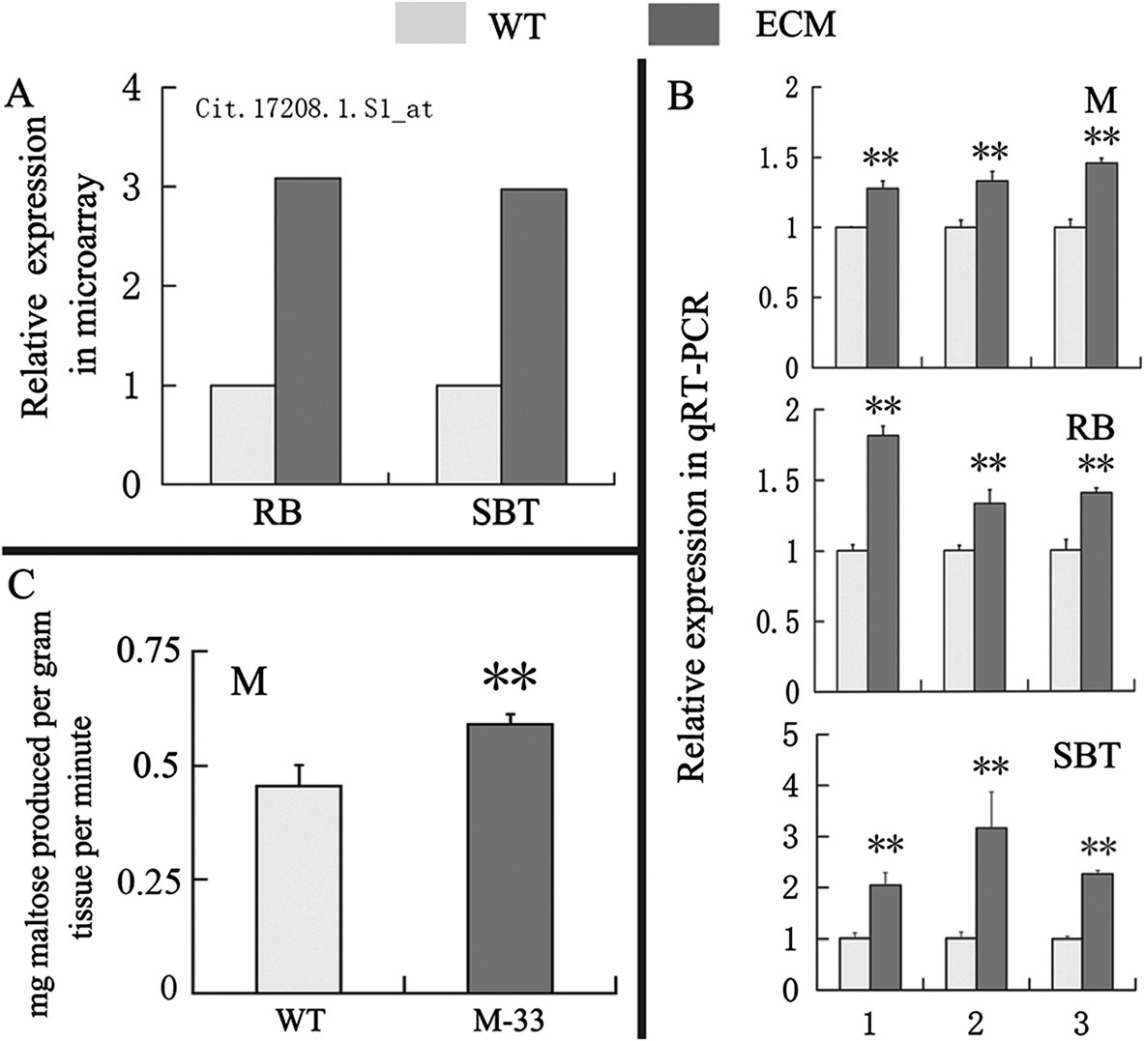
**Validation of the up-regulated transcriptional pattern of the α-amylase gene from microarray data via qRT-PCR and enzyme activity analysis. (A)** Up-regulated transcriptional pattern of Cit.17208.1.S1_at in microarray data, and this gene was annotated as α-amylase gene. **(B)** qRT-PCR analysis of α-amylase genes in M, RB, and SBT. Three α-amylase genes used for qRT-PCR analysis were from a previous report [25] and the NCBI. *AMY*, citrus sinensis alpha-amylase-like gene (accession number: XM_006473264); *SD1*, α-amylase gene (accession number: JN793456); *SD2,* α-amylase 3 gene (accession number: JN793457). Transcript levels are expressed relative to WT (wild type). **(C)** Enzyme activity analysis of α-amylase in wild-type M and M-33. α-amylase activity was expressed as mg maltose produced per gram tissue per minute. Columns and bars represent the means and ± SD, respectively (n = 3 replicate experiments). **Indicates that the values are significantly different compared with wild type at the significance level of P < 0.01. M, RB, and SBT represent Marsh grapefruit, Star Ruby grapefruit, and Sunburst mandarin, respectively. M-33 represents the ECM line of Marsh grapefruit.

MapMan Bin indicated that the three representative ECMs had similar transcriptional responses to carotenoid accumulation (Figure 1). We further examined the differentially expressed genes that were annotated as being involved in stress, redox, hormone metabolism, and secondary metabolism; these groups represented the major transcriptional responses in the ECMs. As shown in Additional file 3, many stress- and redox-response genes were up-regulated in the ECMs. Hormone metabolism genes for ABA, auxin, ethylene, gibberellin, jasmonate (JA), and salicylic acid (SA) showed significantly higher transcription levels in the ECMs than in the wild types. Moreover, many genes involved in the synthesis of phenylpropanoids, alkaloids, wax, and simple phenols were up-regulated in the ECMs, whereas the majority of the genes related to flavonoid/anthocyanin and isoprenoid synthesis were down-regulated. Notably, a MapMan Bin for major CHO metabolism contained one gene that encoded the α-amylase. This gene was up-regulated in the ECMs compared with the wild types (Figure 2A). This result was supported by qRT-PCR analysis and was in accordance with the consistently up-regulated transcriptional pattern of three α-amylase genes (Figure 2B), and their significantly increased enzymatic activity, as shown by enzyme activity analysis (Figure 2C).

Furthermore, gene annotation revealed that in the three ECMs, a significant number of up-regulated genes were annotated as encoding peroxidases (PODs), glutathione S-transferase (GST), and hydroxyproline-rich glycoprotein family proteins. The down-regulated genes primarily encoded protein kinases, zinc finger family proteins, glycine-rich proteins, and senescence-related factors (Additional files 1 and 4).

### Redox status was significantly altered in the ECMs

**Figure 3:**
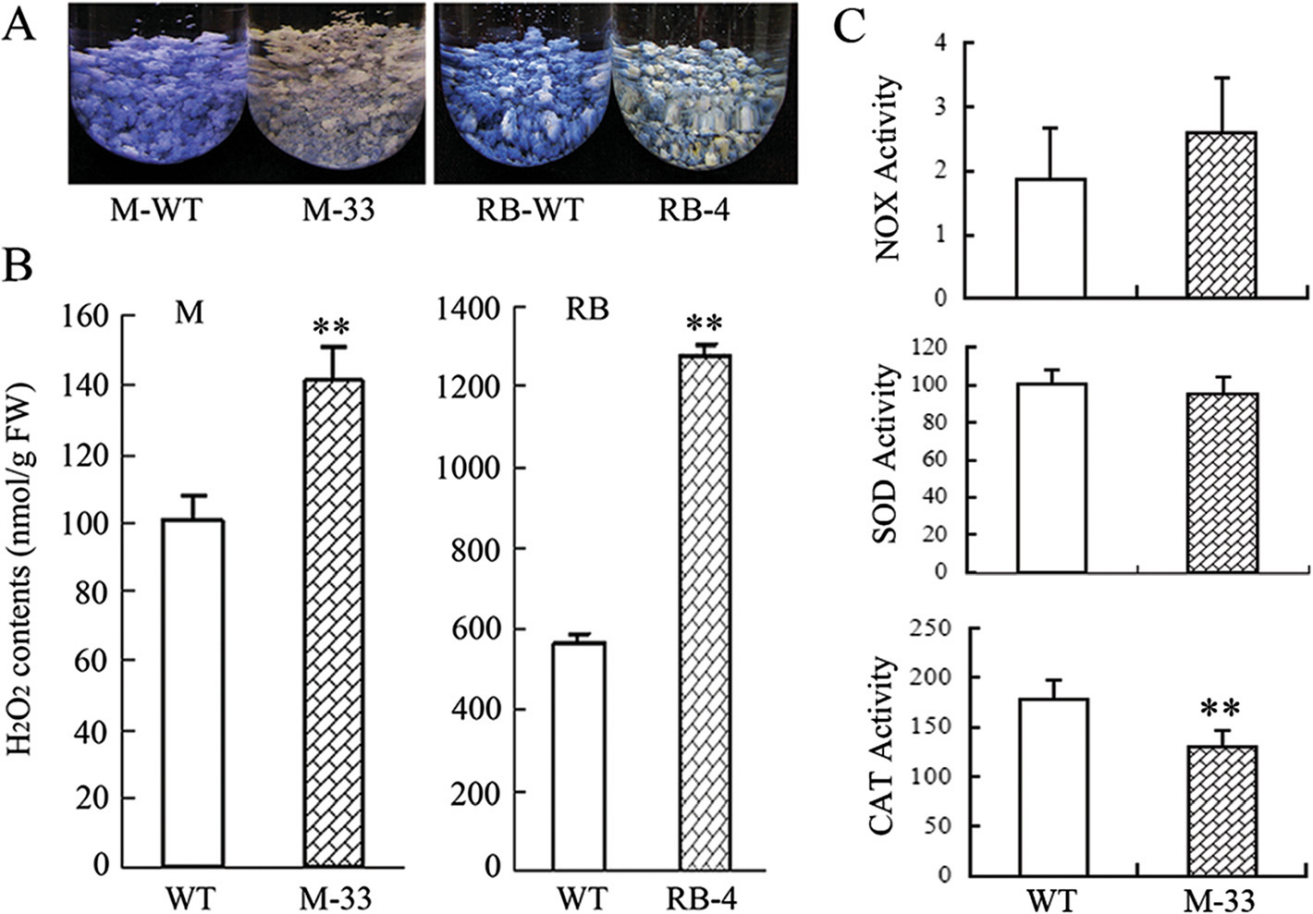
**Determination of ROS levels and the activities of related enzymes in calli.** M-33, and RB-4 represent the ECM lines of Marsh grapefruit and Star Ruby grapefruit, respectively. WT represents wild-type calli. **(A)** O_2_^-^ is detected by histochemical staining with NBT. M and RB are shown as representatives for O_2_^-^ levels. **(B)** H_2_O_2_ determination is based on the titanium reagent method. **(C)** NOX activity (nmol NADPH/min/g FW), SOD activity (U/g FW) and CAT activity (U/g FW) were analyzed in the M-33 and its wild-type control. The columns and bars represent the means and ± SD, respectively (n = 3 replicate experiments). **Indicates that the values are significantly different compared with wild type at the significance level of *P* < 0.01.

POD, GST, hydroxyproline-rich glycoprotein family proteins, heat shock proteins, and universal stress protein (USP) family proteins have often been regarded as important stress response factors in plants [29-31]. Interestingly, our microarray showed that these stress-related genes, especially *PODs*, were significantly induced in the ECMs (Additional files 1 and 4). Prediction of subcellular localization and class of the differentially expressed PODs suggested that they were extracellular class III peroxidases (Additional file 5). Engineered carotenoid modification can disturb ABA levels [32], which could be associated with the stress and redox responses observed in the ECMs. However, comparative analysis showed that, compared with the corresponding wild type, ABA content was higher only in the RB ECM and not in the M and SBT ECMs (Additional file 6). Furthermore, several differentially expressed genes from the microarray data were verified by RT-PCR analysis in the calli, these genes included WRKY75, a protease inhibitor gene, a hydroxyproline-rich glycoprotein family protein gene, and a USP family protein gene (Additional file 7). Previous reports have shown that these four investigated genes are induced by ROS [29-31,33,34]. This information suggested the possibility that engineered carotenoid accumulation modified the ROS levels in the ECMs. Therefore, we investigated the ROS levels in the calli. NBT staining showed that superoxide radical (O_2_^-^) levels in the ECMs were markedly reduced compared with the wild types (Figure 3A). In contrast, hydrogen peroxide (H_2_O_2_) levels showed an unexpected increase (Figure 3B). This result corroborated the microarray data, which revealed that many stress- and redox-responsive genes were up-regulated; H_2_O_2_ has a signaling role in stress responses [29].

To further identify ROS changes in the ECMs, we used M-33 and its wild-type control to analyze the activities of ROS-related enzymes, including NADPH oxidase (NOX), which is requried for O_2_^-^ production, as well as superoxide dismutase (SOD) and catalase (CAT), which are involved in H_2_O_2_ production and scavenging, respectively. As shown in Figure 3C, NOX activity was slightly elevated in M-33, but the difference was statistically insignificant. Similarly, no significant difference in SOD activity was observed between M-33 and the wild-type control. It is noteworthy that CAT activity was significantly lower in M-33 compared with the wild type.

### Carotenoid accumulation altered starch metabolism in ECMs

**Figure 4:**
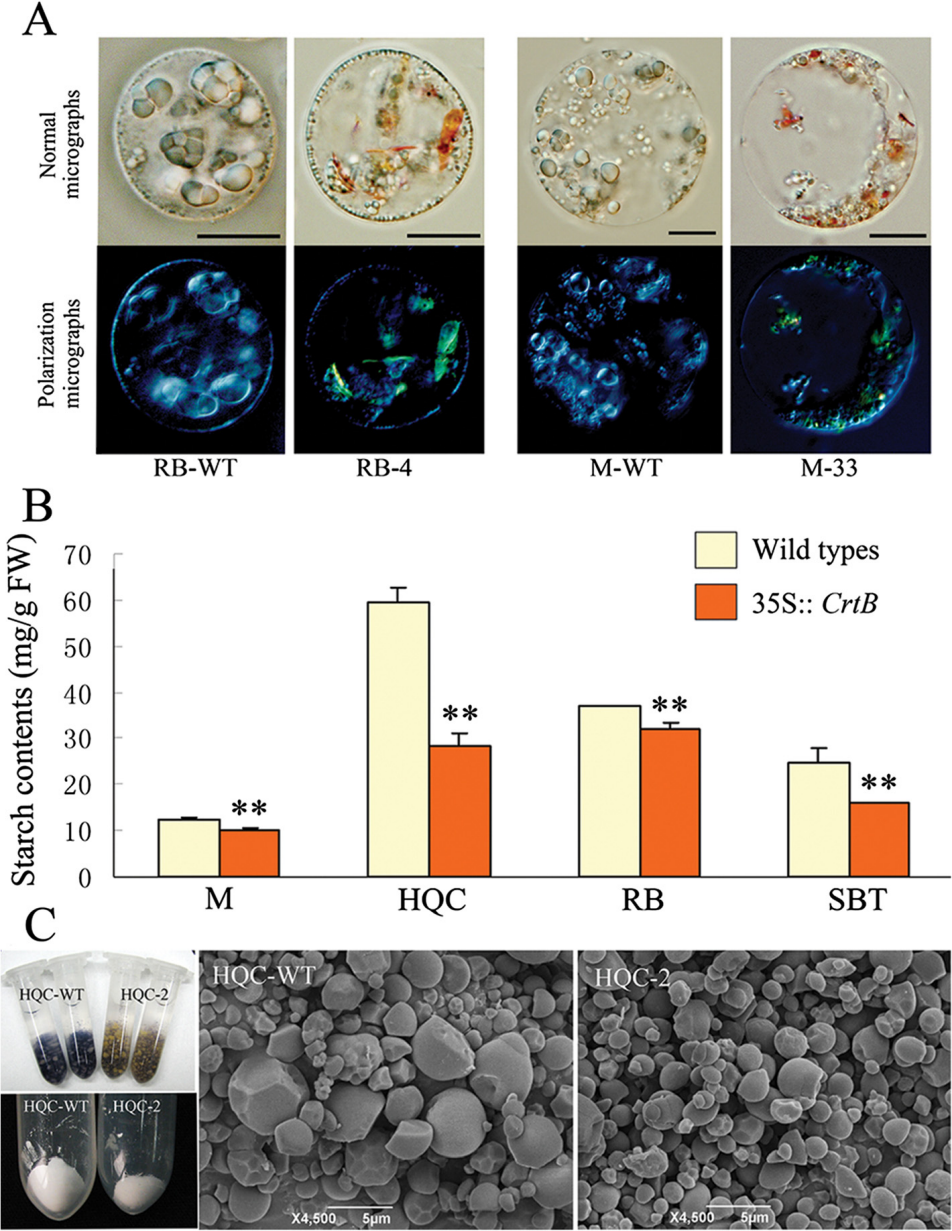
**Comparison of starch accumulation between the ECMs and wild-type calli. (A)** Light microscopic inspection of starch granule deposition in the callus cells. Protoplasts from wild-type calli and representative ECMs, respectively, are observed under a normal light field and a polarization microscope. The polarization microscope shows the characteristic birefringences of starch granules and carotenoid crystals, respectively. **(B)** Starch content analysis. Columns and bars represent the means and ± SD, respectively (n = 3 technical triplicate experiments). **Indicates that the values are significantly different compared with ECMs at the significance level of *P* < 0.01. **(C)** I_2_/KI staining analysis and isolation of starch granules for SEM inspection. HQC is shown as a representative. The image in the lower left corner shows the yields of starch granules from equal weight of calli. HQC-WT and HQC-2 represent the ECM line and wild type, respectively. The right images show the starch granule morphology magnified 4500 times via SEM.

The clear induction of the α-amylase genes and their elevated enzymatic activity (Figures 1 and 2) suggested a modification of starch metabolism in the ECMs. This provided a key clue in explaining some of the phenomena observed in the ECMs. For example, they generally have reduced dry weight (Additional file 8), and their cytological profiles often showed fewer starch granules than the wild types (Figure 4A). Therefore, we investigated starch levels in the calli. Compared with the corresponding wild types, the ECMs had reduced starch content (Figure 4B), which was consistent with the results of iodine staining (Figure 4C). Furthermore, we isolated the starch granules and noted that there are substantially more in the wild types than in the ECMs (Figure 4C). Subsequent SEM investigation demonstrated the absence of large starch granules in ECM cells (Figure 4C). We also discovered that the ECMs had higher soluble sugar (fructose, glucose, and sucrose) content in the ECMs (Additional file 9), which suggested a biochemical alteration of starch metabolism in the ECMs.

### Reduced starch content occurred in parallel with significant carotenoid accumulation in citrus

**Figure 5:**
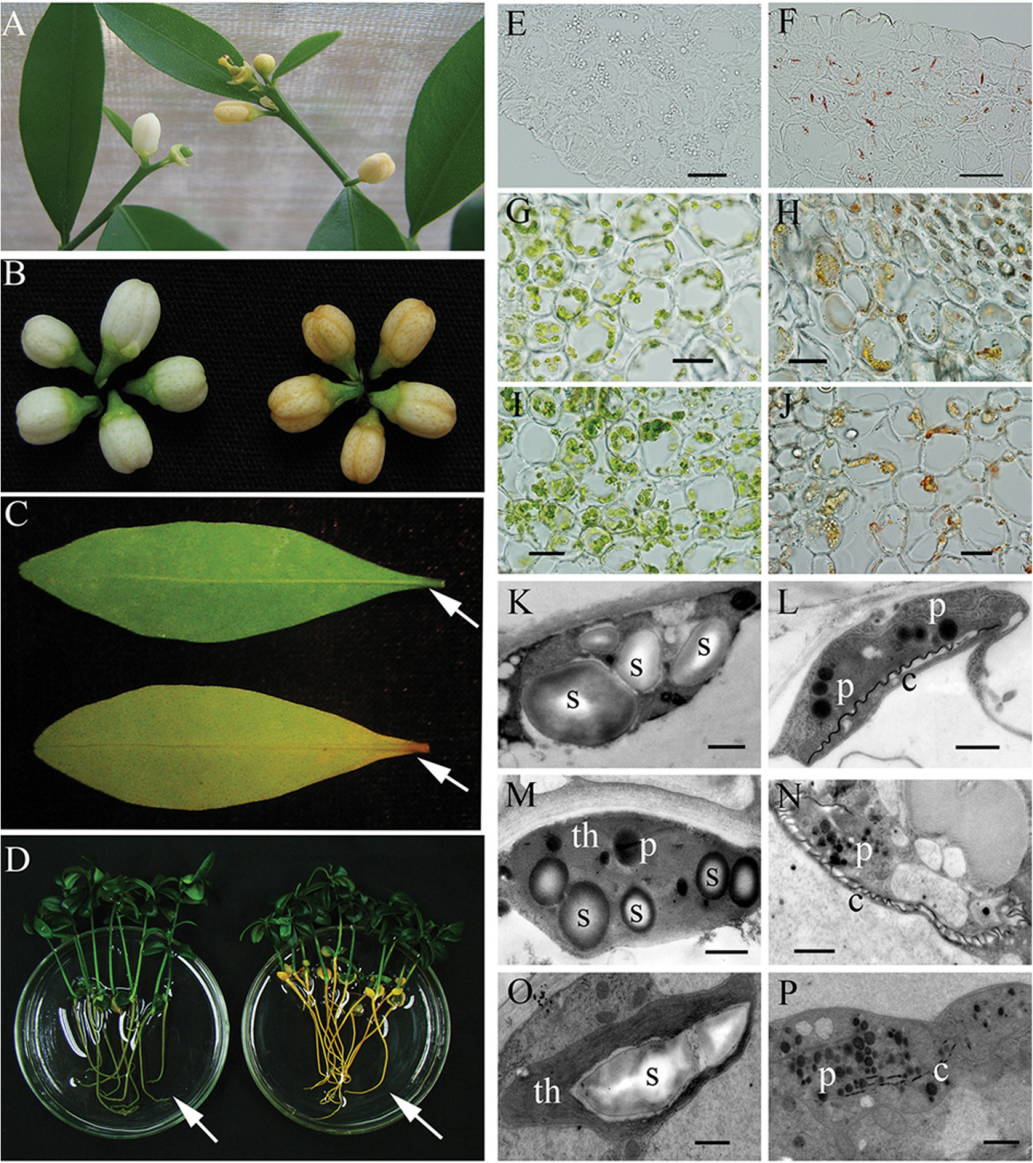
**Phenotypes of transgenic and wild-type Hongkong kumquats and cellular investigation. (A)** Two-year-old flowering Hongkong kumquats (left, wild type; right, 35S:: *CrtB*, which represents transgenic Hongkong kumquat). **(B)** Phenotypes of flowers (left, wild type; right, 35S:: *CrtB*). **(C)** Phenotype of senescent leaf (top, wild type; lower, 35S:: *CrtB*). **(D)** Nucellar seedlings under light-grown for 60 d (left, wild type; right, 35S:: *CrtB*). **(E, G,** and **I)** Frozen sectioning investigation of the petal, leafstalk, and root of the wild-type Hongkong kumquat, respectively. **(F, H,** and **J)** Frozen sectioning investigation of the petal, leafstalk, and root of the transgenic Hongkong kumquat, respectively. **(K, M,** and **O)** Ultrastructural inspection of the petal, leafstalk, and root of the wild-type Hongkong kumquat, respectively. **(L, N,** and **P)** Ultrastructural inspection of petal, leafstalk, and root of the transgenic Hongkong kumquat, respectively. s, starch granules; p, plastoglobules; th, thylakoids; c, carotenoid crystal and characteristic internal membrane. The bars represent 10 μm in light microscopy and represent 1 μm in transmission electron microscopy.

We generated transgenic plants by overexpressing 35S:: *CrtB* in an early flowering citrus relative, Hongkong kumquat (*F. hindsii* Swingle). Overexpression of the *CrtB* gene led to orange pigmentation of the flower petals, roots, and other tissues (Figure 5A-D), which demonstrated increased carotenoid accumulation. Cellular inspection using light and electron microscopy revealed significant modifications of the plastids in the orange tissues, which led the preexisting plastids to show a chromoplast-like profile. For instance, in the wild-type tissues, amyloplasts were found in the flowers and dark-grown roots, etioplasts were found in dark-grown embryoids, and chloroplasts in old petioles and light-grown roots, and all of these plastids showed significant starch granule deposition (Figure 5E, J, I, K, M, O; Additional file 10A). However, in the corresponding transgenic tissues, the plastids showed an entirely different morphology: abundant plastoglobules and crystal structures could be observed, but starch granules and thylakoid membranes were scarce (Figure 5F, H, J, L, N, P; Additional file 10A). We also investigated the expression of genes related to starch metabolism in the roots. The results confirmed that α-amylase genes were significantly up-regulated in transgenic roots (Additional file 10B). These observations demonstrated that the reduction in starch occurred in parallel with significant carotenoid accumulation in citrus, and they also suggested that carotenoid accumulation could induce similar transcriptional perturbation in the roots and in citrus calli. Moreover, six differentially expressed genes involved in stress and senescence that were identified in the microarray data from citrus calli were analyzed in the roots of the transgenic Hongkong kumquat and in the wild-type control. qRT-PCR analysis demonstrated that five of six genes, including those encoding WRKY75, the USP family protein, the hydroxyproline-rich glycoprotein family protein, the senescence-related factor, and the plastocyanin-like domain-containing protein, showed significant transcriptional alterations in the carotenoid-rich roots compared with the wild-type control. Importantly, the changes were consistent with those seen in the ECMs (Additional file 10C).

### Flavonoid/anthocyanin biosynthesis was negatively affected by carotenoid accumulation

**Figure 6:**
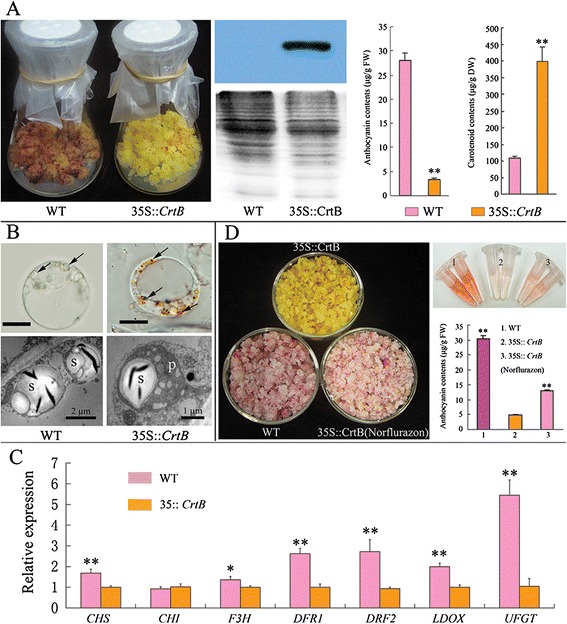
**Pigment accumulation in the transgenic** ***M. hupehensis*** **callus with overexpressive** ***CrtB*** **gene. (A)** Left image shows the phenotypes of the *M. hupehensis* calli cultured under visible light condition; middle image is the western blotting confirmation of CrtB protein, coomassie blue staining for loading control; right image displays the results of pigment (anthocyanins and carotenoids) levels analysis, columns and bars represent the means and ± SD, respectively (n = 3 replicate experiments); 35S:: *CrtB* represents the apple ECM. **(B)** Cytological investigation of *M. hupehensis* callus shows the amyloplasts in the wild-type cell, and the chromoplast-like structures in the ECM cell (arrows shown); s, starch granule; p, plastoglobule; m, mitochondrion. Bars represent 10 μm. **(C)** qRT-PCR analysis of anthocyanin related genes in the *M. hupehensis* calli. UFGT, uridine diphosphate (UDP)-glucose: flavonoid 3-O-glycosyltransferase; LDOX, leucoanthocyanidin dioxygenase; DFR1/2, dihydroflavonol 4-reductase 1/2; F3H, flavanone 3 β-hydroxylase; CHI, chalcone isomerase; CHS, chalcone synthase. Columns and bars represent the means and ± SD, respectively (n = 3 replicate experiments). 35S:: *CrtB* represents the light-cultured apple ECM; WT represents the light-cultured wild-type apple callus. **(D)** Phenotypes and pigment analyses under norflurazon treatment for 20 days. The medium contained 10 μM norflurazon. Columns and bars represent the means and ± SD, respectively (n = 3 replicate experiments). Transcript levels are expressed relative to the apple ECM (35S:: *CrtB*). * and ** indicate that the values are significantly different at the significance levels of P < 0.05 and P < 0.01, respectively.

In the ECMs, transcriptional changes associated with secondary metabolism were obvious (Additional file 3). In particular, the transcription of flavonoid/anthocyanin biosynthetic genes was repressed in the ECMs. Additionally, we tested the expression of chalcone synthase (CHS) and chalcone isomerase (CHI) (the key enzymes of flavonoid biosynthesis) in the roots of the transgenic Hongkong kumquat and its control. qRT-PCR analysis showed that the *CHS* and *CHI* genes were both expressed at lower levels in the orange roots of the transgenic Hongkong kumquats than in the wild-type control (Additional file 10D). These results suggested that carotenoids have a negative effect on flavonoid/anthocyanin accumulation. Citrus embryogenic calli and roots are anthocyanin-free, making it difficult to investigate this phenotype in these tissues. To study this negative correlation, we established an apple ECM by engineering an anthocyanin-rich *M. hupehensis* callus using a 35S:: *CrtB* construct. Overexpression of the *CrtB* gene led to a 3.68-fold increase in total carotenoid levels in the apple ECM (Figure 6A; Additional file 11). Abundant carotenoid accumulation could also be observed through cellular inspection, which showed that apple ECM cells formed many plastoglobules in the plastids, but that the plastids in the wild-type cells were filled with starch granules (Figure 6B). Notably, under visible light, the wild-type callus contained abundant anthocyanins, but the apple ECM showed minimal anthocyanin accumulation (Figure 6A). Additionally, qRT-PCR analysis of anthocyanin biosynthetic genes showed that they were repressed in the apple ECM (Figure 6C). To further understand the negative effect of carotenoids on anthocyanin accumulation in the apple callus system, norflurazon (phytoene desaturase inhibitor) treatment was performed to block colored carotenoid accumulation. After a twenty-day culture with 10 μM norflurazon treatment under visible light, the apple ECM, which was initially yellow, turned red. Anthocyanin analysis revealed that norflurazon treatment could partially rescue anthocyanin accumulation in apple ECM (Figure 6D).

## Discussion

The callus model system has provided a unique opportunity to investigate the effect of carotenoid metabolic engineering in plants [35]. Using our previously constructed, carotenoid-engineered cell models in citrus [28], we examined carotenoid-related biological processes. Despite the genotypic diversity of the representative ECMs, they had similar transcriptional patterns (Figure 1; Additional file 2). Our results revealed new aspects of carotenoid-induced redox modification, starch metabolism, and anthocyanin loss. The present discoveries of carotenoid-related biological processes also support previous studies of carotenoid-associatied plastid development and key metabolic modifications [14,22,23].

### Altered carotenoid accumulation changed the redox status of ECMs

Transcriptomic data showed that *POD* and *GST* genes were significantly induced (Additional file 4). Additionally, genes involved in phenylpropanoid metabolism and hormone metabolism (involved in ABA, JA, and SA) were expressed at higher levels in the ECMs than in the wild types (Additional file 3). These transcriptional characteristics conferred a clear stress response pattern in the ECMs, but this pattern could not be explained by ABA levels (Additional file 6). A previous study revealed that carotenoids can react with ROS to suppress oxidative stress in *Arabidopsis* leaves under high light [7]. In our study, O_2_^-^ levels were markedly reduced in the ECMs compared with the wild types, but NOX and SOD, which are required for O_2_^-^ production and scavenging, respectively, showed insignificant changes in activity in the M-33 ECM (Figure 3A, C). Therefore, it was hypothesized that carotenoids participated in the elimination of O_2_^-^ in the ECMs. However, carotenoid accumulation did not lead to a similar decrease in H_2_O_2_ levels; instead, the ECMs had more H_2_O_2_ than the wild types (Figure 3). This result provided an explanation for the up-regulated stress response in the ECMs, as H_2_O_2_ plays an important signaling role in plant protection systems and could induce a transcriptional pattern similar to that found in the ECMs [29,30]. This hypothesis was supported by qRT-PCR analysis of several ROS-induced genes in the calli (Additional file 7). Increased H_2_O_2_ levels was an unexpected example of ROS modification in the ECMs. One possibility is that significant degradation of O_2_^-^ enhanced H_2_O_2_ production in the ECMs [30,36]. In addition, the reduction of CAT activity in the M-33 ECM provided evidence of a weakened H_2_O_2_ scavenging system, which could lead to increased H_2_O_2_ levels [37].

Similar stress responses were also observed in other plant organs with altered carotenoid accumulation, such as in *OR* transgenic potato and in red-fleshed mutant citrus fruits [16,18]. It is unclear if there is a common mechanism mediating the carotenoid-associated stress response in plants. Recently, carotenoid oxidation products, such as β-cyclocitral, which is generated from the oxidation of β-carotene by singlet oxygen, have been shown to be signals mediating stress responses in *Arabidopsis* [7]. Thus, β-cyclocitral-associated stress response may exist in the ECMs.

### Carotenoid accumulation mediated starch metabolism

Our cytological and biochemical observations (Figure 4 and Additional file 8) reveal a very interesting correlation and suggest that carotenoids might regulate carbohydrate metabolism in plants. The discovery of this process clarifies the developmental processes associated with the nutritional and sensory qualities of agricultural products that accumulate carotenoids. Carotenoid biosynthesis requires a carbohydrate supply for assembling the carotenoid molecular backbone. The plastids are the only organelles involved in carotenoid biosynthesis, and they are also the sites for sugar and starch carbohydrate metabolism [11,18,23]. Thus, a feedback mechanism for maintaining a carbon supply for carotenogenesis could be involved in the correlation between carotenoid accumulation and starch degradation. This hypothesis is supported by a previous investigation that demonstrates a mutually exclusive relationship between carotenoid accumulation and starch deposition during the natural ripening processes of tobacco floral nectaries [19].

In addition, by comparing plastids from white and red carrot roots, Kim *et al*. [22] suggested that carotenoid accumulation might act as a developmental signal directing plastid modification. Carotenogenesis promoted by the overexpression of CrtB or by light has been found to coincide with the differentiation of chromoplasts in carrot roots [38,39]. A recent study further suggests that the adaptation of plastid structures can facilitate the sequestration of the newly formed carotenoids [23]. In the present study, chromoplast-like profiles were observed in various tissues of the 35S:: *CrtB* transgenic *F. hindsii* Swingle, as well as in the ECMs, as reported previously [28]. Therefore, reduced starch content was presumably related to the plastid modification process induced by significant carotenoid accumulation in citrus. This interpretation provides a new perspective to understand the feedback mechanism of carotenogenesis. However, the up-regulation of α-amylase is in apparent contrast with the proteomic analysis of Barsan et al. [11], who showed that proteins involved in starch metabolism decrease in abundance during chromoplastogenesis. Presumably, plastid modifications associated with engineered carotenoid accumulation might involve protein dynamics that differ from those of natural chromoplastogenesis in fruit. Despite the high level of conservation of the chromoplast proteome in the ripening fruits of sweet orange and tomato [40], the plastids in the flower petals, roots, embryoids, petioles, and callus systems of citrus are never involved in chromoplastogenesis during natural developmental processes, and they are distinct from those in citrus and tomato fruits. Additionally, engineered carotenoid accumulation could alter the redox status of ECMs, and this observation suggested that redox-regulated starch degradation occurred in the engineered carotenoid-rich tissues [41]. Further studies are required to identify the direct causal link between carotenoid accumulation and starch reducation.

### Carotenoid accumulation negatively regulates anthocyanin biosynthesis

Carotenoids and anthocyanins are both biological pigments and can co-exist in plant tissues. However, there is little to no anthocyanin accumulation in some carotenoid-rich tissues [25,26]. This phenomenon is not absolute, but it seems to be prevalent in nature. For example, the ripening flesh of tomatoes and apricots accumulates abundant carotenoids but has little to no anthocyanins [42,43]. In addition, the negative correlation between the accumulation of carotenoids and anthocyanins was observed in the peels of five apple genotypes [44]. Although carotenoids and anthocyanins show diverse molecular structures and biosynthetic pathways, they perform similar biological functions, including acting as antioxidants, and as attractants for pollinators [24]. Perhaps, the alternative accumulation of pigments represents an evolutionary mechanism to escape functional redundancy. However, to date, there is no evidence supporting this hypothetic evolutionary mechanism. Our present study found many down-regulated flavonoid/anthocyanin genes in ECMs. This phenomenon was also observed in the carotenoid-rich roots of transgenic 35S:: *CrtB F. hindsii* Swingle. These results suggested a potential effect of carotenoids on anthocyanin biosynthesis. Furthermore, we utilized an apple ECM overexpressing the *CrtB* gene to confirm the negative effect of carotenoid accumulation on anthocyanin biosynthesis.

Compared with the wild type control, the carotenoid-rich apple ECM had minimal anthocyanin accumulation. Additionally, norflurazon could partially rescue anthocyanin accumulation in the apple ECM. These results supported the transcriptional data from citrus, indicating a possible negative effect of carotenoids, and especially colored carotenoids, on anthocyanin accumulation. However, it is known that norflurazon treatment can not only inhibit colored carotenoid biosynthesis, but also alter ROS signaling [45]. Therefore, norflurazon treatment analysis also raised a question about the role of redox state in the correlation between carotenoids and anthocyanins. Anthocyanin accumulation is regarded as a positive response to oxidative stress [46]. The accumulation of carotenoids and their related structures, plastoglobules, may provide a more effective approach than anthocyanin accumulation to suppress oxidation. This interpretation is supported by previous studies showing that increasing levels of xanthophylls or plastoglobules could enhance the photooxidative tolerance and reduce anthocyanin accumulation in *Arabidopsis* and apple leaves [47,48]. Moreover, a recent study has probed into anthocyanin biosynthesis with an early redox signaling control upstream of the known transcription factors [49]. qRT-PCR analysis revealed that most the anthocyanin biosynthetic genes were consistently suppressed in the apple ECM, which suggested that there is significant transcriptional regulation involved in the negative effect of carotenoid accumulation on anthocyanin biosynthesis. However, the redox signaling-based regulatory mechanism that could mediate the link between carotenoid accumulation and anthocyanin biosynthesis is still unclear and requires further study. In addition, in strawberry fruit, competitive regulation via the peroxidase FaPRX27 has recently been proposed; FaPRX27 diverts phenolic flux from anthocyanins to lignin [50]. The existence of such competitive regulation in the apple ECM warrants future study.

## Conclusions

Our studies on the transcriptional patterns of citrus calli linked carotenoid accumulation with redox state, starch metabolism, and flavonoid/anthocyanin accumulation. The existence of these physiological processes was further elucidated using biochemical and cytological analyses, as well as genetic manipulation of carotenoid biosynthesis in citrus calli, *F. hindsii* Swingle, and *M. hupehensis* calli. Our findings provide a new perspective on the complexity of carotenoid accumulation and its associated biological processes. For example, we confirmed that significant carotenoid accumulation could induce starch degradation in callus systems and in tissues such as flower petals and roots. The data generated from these model systems provide important information that could promote the understanding of starch metabolism and carotenoid accumulation during the ripening process in other plant systems. Equally importantly, our discoveries have significant implications for carotenoid metabolic engineering by providing the knowledge needed to give close consideration to a wider range of characteristics, such as plant resistance and systematic metabolic modification. In particular, the decreased anthocyanin levels associated with carotenoid accumulation should be avoided in carotenoid metabolic engineered plants. Anthocyanins are an important source of hydrophilic dietary antioxidants, and fruits and vegetables rich in both soluble and lipophilic antioxidants are considered to offer the best health protection [25].

## Methods

### Plant materials

Engineered cell models (ECMs) were established by over-expressing 35S:: *CrtB* (*tp–rbcS–CrtB)* (CrtB protein, phytoene synthase from *Erwinia herbicola*, now known as *Pantoea agglomerans*, containing a Pea rbcS transit peptide) in citrus embryogenic calli [28]. The ECMs and wild-type embryogenic calli were obtained from four citrus genotypes, Star Ruby grapefruit (*C. paradise* Macf.), Marsh grapefruit (*C. paradise* Macf.), Cara Cara navel orange [*C. sinensis* (L.) Osb.], and Sunburst mandarin [*C. reticulata* Blanco × (*C. paradisi* Macf. × *C. reticulata*)] designated as RB, M, HQC and SBT, respectively. The calli were propagated on MT medium in dark and kept at 25 ± 1°C. MT medium, which is typically used for citrus culture in vitro, was prepared according to Murashige and Tucker [51]. Twenty-day-old calli were harvested and used for immediate cellular and biochemical analyses or stored at -80°C for later molecular analysis.

Transgenic Hongkong kumquats (*Fortunella hindsii* Swingle), an early-flower citrus relative, were recovered through *Agrobacterium-*mediated transformation using 35S:: *CrtB* (*tp–rbcS–CrtB)* construct according to the method of Zhang *et al*. [52]. Regenerated resistant shoots were rooted directly on rooting medium (1/2MT medium supplemented with 0.5 mg/L 1-naphthylacetic acid, 0.1 mg/L indolebutyric acid, 25 g/L sucrose, 0.5 g/L activated charcoal, and 8 g/L agar; pH 5.8). Rooted plantlets were transplanted into pots containing commercial substrates with organic matter and were placed in greenhouse facilities. Nucellar seedlings were recovered through cultivating mature seeds of transgenic and wild-type Hongkong kumquats in solidified MT basal medium containing 20 g/L sucrose.

The apple calli were initiated from the young embryo of the *Malus hupehensis* (a wild apple). They can be grown well on MT medium at 25°C under visible light (40 μmol m^-2^ s^-1^) and display a typical character of anthocyanin accumulation, however, it must be supplemented with 0.1 mg · L^-1^ naphthalene acetic acid (NAA) and 0.5 mg · L^-1^ 6-benzylaminopurine (6-BA). The apple calli were used for genetic transformation using a 35S:: *CrtB* (*tp–rbcS–CrtB)* construct as detailed in our previous paper [28]. Explants preparation and transformation were performed according to the citrus callus transformation protocol described by Cao et al. [28] with a minor modification in which the transgenic calli were selected with 20 mg/L kanamycin. Fifteen-day-old calli were harvested and used for immediate cellular and biochemical analyses or stored at -80°C for molecular analysis. The calli used for extraction of carotenoids were lyophilized and stored at -80°C until use.

Norflurazon (an inhibitor of phytoene desaturase, Sigma, St. Louis, MO, USA) treatment with 10 μM norflurazon (dissolved in acetone) was performed on solid medium for apple calli. Control plates received equivalent acetone. After a twenty-day culture at 25°C under visible light (40 μmol m^-2^ s^-1^), the yellow transgenic apple calli turned a red color, then all samples were collected for anthocyanin analysis.

### Quantitative analysis of gene expression

Total RNA of citrus samples was extracted using a modified Trizol extraction protocol, as described previously [53]. Due to high contents of polyphenol compounds, a CTAB protocol was used to extract the total RNA from apple calli according to Hu *et al*. [54]. First-strand cDNA was synthesized from 1 μg of total RNA isolated from calli and roots using the RevertAid M-MuLV KIT (MBI, Lithuania) according to the manufacturer’s instructions. The primer pairs used in the present study were as listed in previous reports or designed using the Primer Express software (Applied Biosystems, Foster City, CA, USA) (Additional file 12). *UBF5* [a suitable reference gene for qRT-PCR analysis using embryogenic callus culture] and *Actin* were used as the endogenous control to normalize expression in citrus calli and the roots of *F. hindsii* Swingle, respectively [15,55]. In apple calli, *MdActin* was used as the endogenous control [56]. qRT-PCR was performed using ABI 7500 Real Time System (PE Applied Biosystems; Foster City, CA, USA).

### Microarray analysis

Affymetrix GeneChip Citrus Genome Arrays (Affymetrix, Santa Clara, CA, USA) were used for detecting transcriptional diversities between wild-type calli and ECMs. For each sample, RNA was extracted from two biological replicates. A total of 10 μg of fragmented cRNA from each sample was used for hybridization. The procedure for GeneChip Citrus Arrays (hybridization, washing, staining, and scanning with a GeneChip Scanner 3000) was followed carefully according to the Affymetrix GeneChip Expression Analysis Technical Manual.

Scanned images from GeneChip Citrus Arrays were analyzed using GeneChip Operating Software (GCOS 1.4; Affymetrix) with its default settings to generate raw data, which were saved as CEL files. The raw data were normalized using a robust multichip analysis approach implemented in the Affy package [57,58]. Analysis of variance (ANOVA) was used to look for significant differences between samples, using transformation and wild type as factors. The probe sets were filtered for a 2-fold or greater change in expression in RB and SBT, then filtered for a 1.5-fold expression level difference in M. Differentially expressed genes were ranked by *P* values, and genes with a *P* value of ≤0.05 were considered differentially expressed at a statistically significant level. Gene annotation was carried out based on similarity scores in BLASTX comparisons against sequences contained in the Harvest: Citrus database (http://harvest.ucr.edu/). Differentially expressed genes were further analyzed using MapMan Bin (http://ppdb.tc.cornell.edu/default.aspx). The subcellular localization of differentially expressed peroxidase genes was predicted using TargetP (http://www.cbs.dtu.dk/services/TargetP/) and SUBA3 (http://suba.plantenergy.uwa.edu.au/). Peroxidase classification was based on PeroxiBase analysis (https://peroxibase.toulouse.inra.fr/tools/peroxiscan.php).

### Starch analysis

Starch contents in various calli (20-day-old) were detected by the anthrone reagent method according to Chen *et al*. [59]. The procedure of starch granules isolation was based on the method described by Ritte *et al*. [60] with minor modifications. Five grams from each callus were mixed with 10 ml extraction buffer [100 mM *N*-2-hydroxyethylpiperazine-*N*-2-ethanesulfonic acid (HEPES)-KOH (pH 8.0), 1 mM ethylenediaminetetraacetic acid (EDTA), and 0.05% (v/v) Triton-X-100] and homogenized for 20 s using a Waring blender. The homogenate was filtered through 3 layers of Micracloth (Calbiochem), and the pooled filtrates were subsequently centrifuged for 5 min at 1000 g. The supernatant is referred to as the soluble fraction. The remaining pellet was then suspended in 5 ml of extraction buffer. The homogenate was centrifuged for 5 min at 1000 g. The supernatant was discarded, and the pellet was suspended in 2 ml of extraction buffer. Subsequently, the starch suspension was layered on the top of a 5 ml cushion consisting of 90% (v/v) Percoll (GE Healthcare Bio-Sciences AB, Uppsala, Sweden) and 10% (v/v) extraction buffer, the mixture was centrifuged for 15 min at 400 g. The pelleted granules were washed twice in extraction buffer, dried under vacuum condition, and stored at –80°C until use.

α-Amylase activity was assayed by testing for the release of reducing sugars from soluble starch according to a previously described method [61] with appropriate modifications. The assay buffer consisted of 50 mM Na-acetate and 10 mM CaCl_2_, pH 5.2. Heat-treated extracts (70°C for 15 min) were used to inactivate β-amylase. The substrate was 1% boiled soluble starch and incubation (40°C) lasted for up to 5 min. An aliquot (200 μl) was taken from the assay mixture, treated with 2 ml of 3,5-dinitrosalicylic acid (DNS) solution (40 mM DNS, 400 mM NaOH, and 1 M K-Na tartrate), then heated for 10 min at 100°C. After dilution with distilled water (up to 5 ml), the A520 was taken, and the reducing power was evaluated using a standard curve obtained using maltose. α-Amylase activity was expressed as mg of maltose produced per gram of tissue per minute.

### Soluble sugar content measurement

Twenty-day-old calli were were washed 5 times using distilled water to remove the soluble sugar from the medium. Soluble sugar contents were quantified using gas chromatography. Two grams of fresh calli were homogenized and reconstituted in 80% (v/v) methanol for 30 min at 70°C. After centrifugation at 4000 g for 10 min, the supernatant was withdrawn and diluted to a volume of 10 ml; 0.2 ml of methyl-*α*-D-glucopyranoside and phenyl β-D-glucopyranoside was added as an internal standard. The procedure for derivatization was performed as described by Bartolozzi *et al*. [62]. The derivatized samples were injected into an Agilent 6890 N gas chromatograph (Agilent, Palo Alto, CA, USA) using an Agilent 7683 autosampler.

### Measurement of ABA levels

Various calli for abscisic acid (ABA) quantification were prepared according to the method described by Pan *et al*. [63] with some modifications. Calli (0.8 g per sample) were ground into powder in liquid nitrogen, and each sample was transferred to 10 ml screw-cap tubes. Two microliters of extraction solvent [2-propanol: H_2_O: concentrated HCl (2: 1: 0.002, v/v/v)] was added to each tube and shaken at 200 rpm for 30 min at 4°C. Subsequently, dichloromethane (4 ml) was added to each sample and the mixture was continually shaken for 30 min at 4°C. The mixtures were centrifuged at 13000 g for 5 min, then the lower phase was transferred into a screw-cap tube and concentrated using the nitrogen. The samples were redissolved in 0.2 ml of methanol and filtered with 0.22 μm organic membrane filters for analysis via HPLC electrospray ionization tandem mass spectrometry (HPLC-ESI-MS/MS). An Agilent 1100 HPLC (Agilent Technologies, Palo Alto, CA, USA), a Waters C18 column (150 × 2.1 mm, 5 μm), and the API3000 MS-MRM (Applied Biosystems, Foster City, CA, USA) were used for ABA measure. The reaction monitoring acquisition of the transition 263/153 was used for quantitation of ABA extracts.

### ROS levels and the activities of related enzymes

Twenty-day-old calli were harvested for ROS analysis. *In situ* accumulation of O_2_^-^ was examined based on histochemical staining by nitroblue tetrazolium (NBT) [64]. H_2_O_2_ determination was based on the fact that hydroperoxides form a specific complex with titanium (Ti4^+^) that can be measured by colorimetry, as described by Brennan and Frekel [65].

NADPH oxidase (NOX, EC 1.6.3.1) activity in the callus samples was determined using a commercial plant NADPH oxidase detection kit (GMS50096.3, Genmed Scientifics Inc. USA) according to the manufacturer’s instructions. Extraction of superoxide dismutase (SOD, EC 1.15.1.1) and catalase (CAT, EC 1.11.1.6) was conducted as previously described [66]. SOD activity was spectrophotometrically measured using a photochemical assay system based on the inhibition of NBT reduction, and one unit of SOD was defined as the enzyme quantity that inhibited NBT photoreduction by 50% [67]. The CAT activity was assessed by monitoring the decrease in absorbance at 240 nm resulting from H_2_O_2_ consumption, and one unit of CAT activity was defined as a 0.01 reduction in absorbance units per min [66].

### Western blot analysis

Total proteins of *M. hupehensis* calli were prepared via the phenol extraction protocol described by Pan *et al*. [16]. The total proteins were quantified using a Bio-Rad protein assay kit (Bio-Rad, Hercules, CA, USA) based on the Lowry method using bovine serum albumin (BSA) as standard. Anti-CrtB antibodies were generated through immunizing rabbits using a peptide that contains 117 amino acids in the C-terminus of CrtB [28]. Subsequent protein separation and Western blot analysis were performed accordingly to Cao *et al*. [28].

### Pigment analyses

Carotenoid extraction and analysis using reversed-phase high-performance liquid chromatography (RP-HPLC) was conducted as previously described [28]. Because of carotenoid esters in *M. hupehensis* calli, the extracts were saponified with 15% (w/v) KOH: methanol. The carotenoids were identified by their characteristic absorption spectra and typical retention time which were based on the literature and standards of the CaroNature Co. (Bern, Switzerland). The quantification of the carotenoids was achieved using calibration curves for violaxanthin, lutein, antheraxanthin, phytoene, α-carotene, β-carotene, and lycopene; phytofluene was quantified as phytoene, luteoxanthin was quantified as lutein, and zeaxanthin was quantified as antheraxanthin.

Total anthocyanins were measured using a spectrophotometric differential pH method following the procedure of Yuan *et al*. [68] with a minor modification. Frozen samples (400 mg) were crushed into powder and extracted separately with 2 ml of pH 1.0 buffer containing 50 mM KCl and 150 mM HCl as well as with 2 ml of pH 4.5 buffer containing 400 mM sodium acetate and 240 mM HCl. The mixtures were centrifuged at 12000 g for 15 min at 4°C. Supernatants were collected and diluted for direct measurement of absorbance at 510 nm. Total anthocyanin content was calculated using the following equation: amount (μg/g FW) = (A_pH1_ - A_pH4.5_) × 1000 × 484.8/24825 × 6. The number 484.8 is the molecular mass of cyanidin-3-glucoside chloride and 24825 is its molar absorptivity at 510 nm. Six is the dilution factor in this experiment.

### Microscopy analyses

Protoplasts from the calli were generated as previously described [69], then protoplast suspensions were dropped onto microscope slides to observe the plastid modes. Light microscopy of various orange tissues of Hongkong kumquats was performed using a frozen sectioning technique with a Leica CM1900 (Leica, Germany). An optical microscope (BX61, Olympus) equipped with a DP70 camera was used in tandem with a differential interference contrast (DIC) technique.

Transmission electron microscopy (TEM) analysis was performed according to Cao *et al*. [28]. Samples were prepared using a normal fixation process with 2.5% glutaraldehyde adjusted to pH 7.4, and a 0.1 M phosphate buffer with 2% OsO_4_. The preparations were dehydrated and embedded in epoxy resin and SPI-812, respectively. Ultrathin sections obtained with a Leica UC6 ultramicrotome were stained with uranyl acetate and subsequently with lead citrate. Image recording was performed with a HITACHI H-7650 transmission electron microscope at 80 KV and a Gatan 832 CCD camera.

Starch granule morphology was examined with a scanning electron microscope (SEM). The samples were mounted on studs, sputter coated with gold (Balzers, JFC-1600), and examined under a JSM-6390LV SEM (JEOL, Japan).

### Statistical analysis

The SAS statistical software was used to compare the statistical difference based on the Student-Newman-Keuls’ multiple range test at significance levels of *P* < 0.05 (*) and *P* < 0.01 (**), respectively. A linear regression calculation was implemented in a Microsoft Excel® spreadsheet.

### Availability of supporting data

The raw data sets supporting the results of this article are available in the Gene Expression Omnibus (GEO) repository under accession No. GSE61633 at website: http://www.ncbi.nlm.nih.gov/geo/query/acc.cgi?acc = GSE61633, and LabArchives (doi:10.6070/H4XW4GRZ).

## Additional files

Additional file 1:
**List of differentially expressed genes (including annotation and MapMan Bin) between the wild types and ECMs.**
Additional file 2:
**Validation of the microarray expression data using qRT-PCR.** **(A)** Relative transcript levels of 10 genes; M, RB, and SBT represent Marsh grapefruit, Star Ruby grapefruit, and Sunburst mandarin, respectively. Transcript levels are expressed relative to WT (wild type). **(B)** Microarray probes used for qRT-PCR validation and the annotation. **(C)** Comparison between the gene expression ratios obtained from microarray data and qRT-PCR. Additional file 3:
**Number of differentially expressed genes involved in stress and redox, hormone metabolism, and secondary metabolism.** M, RB, and SBT represent Marsh grapefruit, Star Ruby grapefruit, and Sunburst mandarin, respectively. Positive axes represent the number of genes up-regulated, and negative axes represent the number of genes down-regulated. Additional file 4:
**Function annotation of predominantly detected genes.** The graphs show number of genes annotated as the same function. The predominant functions are listed on the right of the graphs. M, RB, and SBT represent Marsh grapefruit, Star Ruby grapefruit, and Sunburst mandarin, respectively. Additional file 5:
**Subcellular localization and class of differentially expressed peroxidase genes in this study.**
Additional file 6:
**ABA contents in the ECMs and their wild-type controls.** Columns and bars represent the means and ± SD, respectively (n = 3 replicate experiments). **indicates that the values are significantly different compared with wild type at the significance level of P < 0.01. M-33, RB-4, and SBT-6 represent the ECM lines of Marsh grapefruit, Star Ruby grapefruit, and Sunburst mandarin, respectively. Additional file 7:
**Differentially expressed ROS-induced genes from the microarray data were verified in the calli via RT-PCR analysis.** M, RB, and SBT represent Marsh grapefruit, Star Ruby grapefruit, and Sunburst mandarin, respectively. Trangenic calli (35S:: *CrtB*) were the representative ECMs, M-33, RB-4, and SBT-6, which were also used for Affymetrix microarray analysis. Additional file 8:
**Dry weight analysis of the wild types and ECMs.** Columns and bars represent the means and ± SD, respectively (n = 3 biological replicate experiments. **indicates that the values are significantly different at the significance level of P < 0.01. Additional file 9:
**Soluble sugar (fructose, glucose, and sucrose) contents in the ECMs and wild types.** 35S:: *CrtB* represents the ECM lines. RB, M, HQC, and SBT represent Star Ruby grapefruit, Marsh grapefruit, Cara Cara navel orange, and Sunburst mandarin, respectively. Columns and bars represent the means and ± SD, respectively (n = 3 biological replicate experiments). * and ** indicate that the values are significantly different compared with wild type at the significance levels of P < 0.05 and P < 0.01, respectively. Additional file 10:
**Cellular investigation and qRT-PCR analysis of the roots of Hongkong kumquats (** ***F. hindsii*** **Swingle).** **(A)** Cellular investigation of Hongkong kumquats. Ultrastructural inspection of dark-grown roots and embryoids. 35S:: *CrtB* represents the transgenic line. s, starch granules; p, plastoglobules; th, thylakoids; c, carotenoid crystal and characteristic internal membrane; ch, chromoplast; am, amyloplast. **(B)** qRT-PCR analysis of starch related genes in the roots of Hongkong kumquats. AMY, citrus sinensis alpha-amylase-like; SD1, α-amylase; SD2*,* α-amylase 3. **(C)** Expression levels of 6 stress-related and senescence-related genes that had been identified as differentially expressed between the ECMs and their wild types in microarray and qRT-PCR analyses. 1, WRKY75 (Cit.341.1.S1_s_at); 2, Protease inhibitor (Cit.16616.1.S1_at); 3, Universal stress protein (USP) family protein (Cit.14892.1.S1_at); 4, Hydroxyproline-rich glycoprotein family protein (Cit.37479.1.S1_at); 5, Senescence-related gene (Cit.14916.1.S1_at); 6, Plastocyanin-like domain-containing protein (Cit.5498.1.S1_at). **(D)** qRT-PCR analysis of key flavonoid biosynthetic genes in the roots of transgenic Hongkong kumquat and its control. CHS, chalcone synthase; CHI, chalcone isomerase. All transcript levels are expressed relative to WT (wild type). * and ** indicate that values are significantly different compared with wild type at the significance levels of *P* < 0.05 and *P* < 0.01, respectively. Additional file 11:
**Contents of various carotenoids in the** ***M. hupehensis*** **calli.** Vio., Violaxanthin; Luteo., Luteoxanthin; Lut., lutein; Phy., Phytoene; Phytof., Phytofluene; Anth., Antheraxanthin; Zea., Zeaxanthin; β-Car., β-Carotene; Lcy., Lycopene. WT represents the light-cultured wild-type apple callus and 35S:: *CrtB* represents the light-cultured transgenic apple callus with overexpression of *CrtB*. Columns and bars represent the means and ± SD, respectively (n = 2 replicate experiments). * and ** indicate that the values are significantly different compared with wild type at the significance levels of *P* < 0.05 and *P* < 0.01, respectively. Additional file 12:
**Specific primer pairs used for qRT-PCR analysis in the present study.**
