# Supplementary material for: Carotenoid accumulation affects redox status, starch metabolism, and flavonoid/anthocyanin accumulation in citrus
Source: BMC Plant Biol. 2015 Feb 3;15:27. doi: 10.1186/s12870-015-0426-4 (PMC4323224; doi:10.1186/s12870-015-0426-4)
Supplement: Additional file 2: — Validation of the microarray expression data using qRT-PCR. (A) Relative transcript levels of 10 genes; M, RB, and SBT represent Marsh grapefruit, Star Ruby grapefruit, and Sunburst mandarin, respectively. Transcript levels are expressed relative to WT (wild type). (B) Microarray probes used for qRT-PCR validation and the annotation. (C) Comparison between the gene expression ratios obtained from microarray data and qRT-PCR. [file 12870_2015_426_MOESM2_ESM.pdf]

Additional material

Additional File 2. Specific primer pairs used for qRT-PCR analysis in the present study.

Gene detected	Sequence
Validation of microarray data	
Cit.1332.1.S1_s_at	Forward: 5'-CACCCCCGCCACCAA-3'
	Reverse: 5'-GTATCCGATGCCGTTAGTGTTTT-3'
Cit.14916.1.S1_at	Forward: 5'-CCTTTCGGTAGCAGCATTTCA-3'
	Reverse: 5'-TCCACGGAATTTGGTAAAGGA-3'
Cit.13606.1.S1_at	Forward: 5'-TGCGCTGATATTTTAGCCATTG-3'
	Reverse: 5'-GGCCCTCCAAGAATTGCA-3'
Cit.22157.1.S1_at	Forward: 5'-CAATTCCTTCCATCCCTTTCTTC-3'
	Reverse: 5'-CAAGTGACAAATTCAAGGGCTCTT-3'
Cit.4353.1.S1_s_at	Forward: 5'-GCAGCAGCCTCGCATCA-3'
	Reverse: 5'-GCCTGGGTGCTTGGTCAGT-3'
Cit.5498.1.S1_at	Forward: 5'-GCATGTTCCTGCAGATGAAGTG-3'
	Reverse: 5'-GGCCGATGGCTGTGCTT-3'
Cit.13114.1.S1_s_at	Forward: 5'-CGGCTAGAGGCTGGAAAGG-3'
	Reverse: 5'-ACTGGCCTAGGGTTCATGAAGA-3'
Cit.38640.1.S1_at	Forward: 5'-TGTCAAGTCCAAACTCCACCAA-3'
	Reverse: 5'-CGAGGTTGCTGGTCCATTG-3'
Cit.1558.1.S1_at	Forward: 5'-GGTTGCAGCAGCTGGATTCT-3'
	Reverse: 5'-CATTTGGATCCCCATGAAAGA-3'
Cit.10215.1.S1_s_at	Forward: 5'-GAGATTGAAGGGAAGTTCGTGAA-3'
	Reverse: 5'-CGGCGTTCTCCTCCAAGTAC-3'
Cit.341.1.S1_s_at	Reverse: 5'-TCACACCATCCATCAAACTCTTAAG-3'
	Reverse: 5'-GTTGCAGTTGTGGGTTCCAA-3'
Cit.16616.1.S1_at	Reverse: 5'-CTCCAGCCGATTCTTCAGATG-3'
	Reverse: 5'-CTGAAGGGATGCTTGGCATT-3'
Cit.14892.1.S1_at	Reverse: 5'-AGGCACGTACGGTTGGTGTT-3'
	Reverse: 5'-GAGGGCTGCTTTGCTGTTG-3'
Cit.37479.1.S1_at	Reverse: 5'-CCGTTGCCCTCAATCCAAT-3'
	Reverse: 5'-TTGTGGAGGGAATGTTTGGAA-3'
qRT-PCR analysis of α-amylase, chalcone synthase, and chalcone isomerase related genes in citrus	
*AM*Y	Forward: 5'-CAAGTAACAAGGCAGGAGGATG-3'
	Reverse: 5'-GGATTGAGACGGTGGAGGAA-3'
SD1 *	Forward: 5'-GGTATCCTCCAAGCTGCTGTG-3'
	Reverse: 5'-ACTTTATCCGATGGGAATGGC-3'
SD2 *	Forward: 5'-AAGGAATAAAATCCACTGCCGTAG-3'
	Reverse: 5'-CTTGGAGGTTCATAATGACCTGGT-3'
CHS	Forward: 5'-CGATGGTGCGGCTGCTAT-3'
	Reverse: 5'-GGCTTCTCGATCTCGGGTATC-3'
CHI	Forward: 5'-GAGATTGAAGGGAAGTTCGTGAA-3'
	Reverse: 5'-CGGCGTTCTCCTCCAAGTAC-3'
Actin [@]	Forward: 5'-AACCGAACGTGCCCATTT-3'
	Reverse: 5'-TGTCTACGAACACCGCATACTT-3'
UBF5 [$]	Forward: 5'- CAAGGACTACGAGATTCACGAC-3'
	Reverse: 5'- CCTGCCGCATCTTCCAAT-3'
qRT-PCR analysis of anthocyanin related genes in apple	
MdActin [#]	Forward: 5'-TGACCGAATGAGCAAGGAAATTACT-3'
	Reverse: 5'-TACTCAGCTTTGGCAATCCACATC-3'

UFGT #	Forward: 5’-CCACCGCCCTTCCAAACACTCT-3’
	Reverse: 5’-CACCCTTATGTTACGCGGCATGT-3’
LDOX #	Forward: 5’-CCAAGTGAAGCGGGTTGTGCT-3’
	Reverse: 5’-CAAAGCAGGCGGACAGGAGTAGC-3’
DFR2 #	Forward: 5’-GATAGGGTTTGAGTTCAAGTA-3’
	Reverse: 5’-CGGCCGGAATCGGAATCAATC-3’
DFR1 #	Forward: 5’-GATAGGGTTTGAGTTCAAGTA-3’
	Reverse: 5’-TCTCCTCAGCAGCCTCAGTTTTCT-3’
F3H #	Forward: 5’-TGGAAGCTTGTGAGGACTGGGGT-3’
	Reverse: 5’-CTCCTCCGATGGCAAATCAAAGA-3’
CHI #	Forward: 5’-GGGATAACCTCGCGGCCAAA-3’
	Reverse: 5’-GCATCCATGCCGGAAGCTACAA-3’
CHS #	Forward: 5’-GGAGACAACTGGAGAAGGACTGGAA-3’
	Reverse: 5’-CGACATTGATACTGGTGTCTTCA-3’

Primers marked with * are from Liao et al. (2012); primers marked with @ are from Liu et al. (2007); primers marked with $ are from Liu et al. (2013); primers marked with # are from Espley et al. (2013); other primers were designed using the Primer Express software (Applied Biosystems, Foster City, CA, USA). *AMY*, citrus sinensis alpha-amylase-like (accession number: XM_006473264); *SD1*, α-amylase (accession number: JN793456); *SD2*, α-amylase 3 (accession number: JN793457). UFGT, uridine diphosphate (UDP)-glucose: flavonoid 3-O-glycosyltransferase; LDOX, leucoanthocyanidin dioxygenase; DFR1/2, dihydroflavonol 4-reductase 1/2; F3H, flavanone 3β-hydroxylase; CHI, chalcone isomerase; CHS, chalcone synthase.
