# Supplementary material for: Carotenoid accumulation affects redox status, starch metabolism, and flavonoid/anthocyanin accumulation in citrus
Source: BMC Plant Biol. 2015 Feb 3;15:27. doi: 10.1186/s12870-015-0426-4 (PMC4323224; doi:10.1186/s12870-015-0426-4)
Supplement: Additional file 3: — Number of differentially expressed genes involved in stress and redox, hormone metabolism, and secondary metabolism. M, RB, and SBT represent Marsh grapefruit, Star Ruby grapefruit, and Sunburst mandarin, respectively. Positive axes represent the number of genes up-regulated, and negative axes represent the number of genes down-regulated. [file 12870_2015_426_MOESM3_ESM.pdf]

Additional File 3. Subcellular localization and class of differentially expressed peroxidase genes in this study.

Genotype	Probeset ID	Arabidopsis Accession	E-value	Gene description	Prediction of localization		Class of peroxidase
					Targetp	SUBA	
M	Cit.26455.1.S1_at	AT5G67400.1	9.00E-61	peroxidase 73 (PER73) (P73) (PRXR11)	Extracellular	Extracellular	Class III peroxidase
	Cit.13855.1.S1_at	AT1G68850.1	1.00E-130	peroxidase, putative	Extracellular	Extracellular	Class III peroxidase
	Cit.30798.1.S1_at	AT5G05340.1	1.00E-95	peroxidase, putative	Extracellular	Extracellular	Class III peroxidase
	Cit.1827.1.S1_s_at	AT5G06730.1	1.00E-117	peroxidase, putative	Extracellular	Extracellular	Class III peroxidase
	Cit.1827.1.S1_at	AT5G06730.1	1.00E-117	peroxidase, putative	Extracellular	Extracellular	Class III peroxidase
	Cit.13606.1.S1_at	AT5G58390.1	5.00E-83	peroxidase, putative	Extracellular	Extracellular	Class III peroxidase
RB	Cit.858.1.S1_s_at	AT1G71695.1	1.00E-118	peroxidase 12 (PER12) (P12) (PRXR6)	Extracellular	Extracellular	Class III peroxidase
	Cit.16394.1.S1_at	AT5G66390.1	1.00E-116	peroxidase 72 (PER72) (P72) (PRXR8)	Extracellular	Extracellular	Class III peroxidase
	Cit.26455.1.S1_at	AT5G67400.1	9.00E-61	peroxidase 73 (PER73) (P73) (PRXR11)	Extracellular	Extracellular	Class III peroxidase
	Cit.13855.1.S1_at	AT1G68850.1	1.00E-130	peroxidase, putative	Extracellular	Extracellular	Class III peroxidase
	Cit.11855.1.S1_s_at	AT5G05340.1	1.00E-103	peroxidase, putative	Extracellular	Extracellular	Class III peroxidase
	Cit.30798.1.S1_at	AT5G05340.1	1.00E-95	peroxidase, putative	Extracellular	Extracellular	Class III peroxidase
	Cit.8515.1.S1_s_at	AT5G06720.1	1.00E-116	peroxidase, putative	Extracellular	Extracellular	Class III peroxidase
	Cit.8515.1.S1_x_at	AT5G06730.1	1.00E-106	peroxidase, putative	Extracellular	Extracellular	Class III peroxidase
	Cit.13606.1.S1_at	AT5G58390.1	5.00E-83	peroxidase, putative	Extracellular	Extracellular	Class III peroxidase
	Cit.8282.1.S1_s_at	AT1G05260.1	1.00E-123	RCI3 (RARE COLD INDUCIBLE GENE 3); peroxidase	Extracellular	Extracellular	Class III peroxidase
SBT	Cit.16394.1.S1_at	AT5G66390.1	1.00E-116	peroxidase 72 (PER72) (P72) (PRXR8)	Extracellular	Extracellular	Class III peroxidase
	Cit.26455.1.S1_at	AT5G67400.1	9.00E-61	peroxidase 73 (PER73) (P73) (PRXR11)	Extracellular	Extracellular	Class III peroxidase
	Cit.13855.1.S1_at	AT1G68850.1	1.00E-130	peroxidase, putative	Extracellular	Extracellular	Class III peroxidase
	Cit.30798.1.S1_at	AT5G05340.1	1.00E-95	peroxidase, putative	Extracellular	Extracellular	Class III peroxidase
	Cit.11855.1.S1_s_at	AT5G05340.1	1.00E-103	peroxidase, putative	Extracellular	Extracellular	Class III peroxidase
	Cit.23268.1.S1_s_at	AT5G05340.1	1.00E-110	peroxidase, putative	Extracellular	Extracellular	Class III peroxidase
	Cit.8509.1.S1_s_at	AT5G06720.1	1.00E-113	peroxidase, putative	Extracellular	Extracellular	Class III peroxidase
	Cit.8515.1.S1_s_at	AT5G06720.1	1.00E-116	peroxidase, putative	Extracellular	Extracellular	Class III peroxidase
	Cit.1827.1.S1_s_at	AT5G06730.1	1.00E-117	peroxidase, putative	Extracellular	Extracellular	Class III peroxidase
	Cit.1827.1.S1_at	AT5G06730.1	1.00E-117	peroxidase, putative	Extracellular	Extracellular	Class III peroxidase
	Cit.13606.1.S1_at	AT5G58390.1	5.00E-83	peroxidase, putative	Extracellular	Extracellular	Class III peroxidase
	Cit.16262.1.S1_at	AT1G05260.1	3.00E-20	RCI3 (RARE COLD INDUCIBLE GENE 3); peroxidase	Extracellular	Extracellular	Class III peroxidase

Prediction of subcellular localization were carried out using TargetP (http://www.cbs.dtu.dk/services/TargetP/) and SUBA3 (http://suba.plantenergy.uwa.edu.au/). Class of peroxidase was based on the PeroxiBase analysis (https://peroxibase.toulouse.inra.fr/tools/peroxiscan.php).
