# Supplementary material for: Carotenoid accumulation affects redox status, starch metabolism, and flavonoid/anthocyanin accumulation in citrus
Source: BMC Plant Biol. 2015 Feb 3;15:27. doi: 10.1186/s12870-015-0426-4 (PMC4323224; doi:10.1186/s12870-015-0426-4)
Supplement: Additional file 4: — Function annotation of predominantly detected genes. The graphs show number of genes annotated as the same function. The predominant functions are listed on the right of the graphs. M, RB, and SBT represent Marsh grapefruit, Star Ruby grapefruit, and Sunburst mandarin, respectively. [file 12870_2015_426_MOESM4_ESM.pdf]

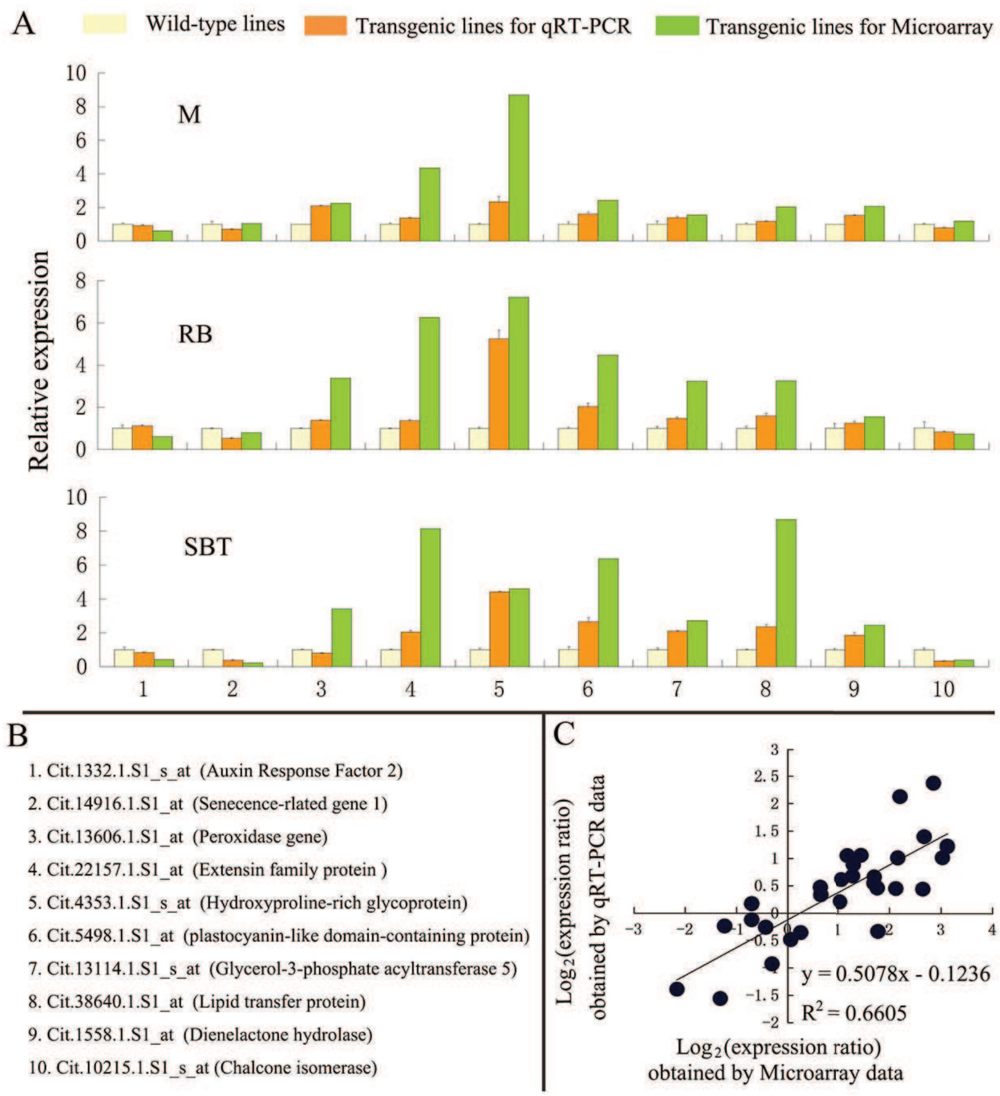

Additional File 4. Validation of the microarray expression data using qRT-PCR. (A) Relative transcript levels of 10 genes; M, RB, and SBT represent Marsh grapefruit, Star Ruby grapefruit, and Sunburst mandarin, respectively. Trangenic callus lines were the representative ECMs, M-33, RB-4, and SBT-6, which were also used for Affymetrix microarray analysis. Transcript levels are expressed relative to WT (wild type). (B) Microarray probes used for qRT-PCR validation and the annotation. (C) Comparison between the gene expression ratios obtained from microarray data and qRT-PCR.
