# Supplementary material for: Carotenoid accumulation affects redox status, starch metabolism, and flavonoid/anthocyanin accumulation in citrus
Source: BMC Plant Biol. 2015 Feb 3;15:27. doi: 10.1186/s12870-015-0426-4 (PMC4323224; doi:10.1186/s12870-015-0426-4)
Supplement: Additional file 5: — Subcellular localization and class of differentially expressed peroxidase genes in this study. [file 12870_2015_426_MOESM5_ESM.pdf]

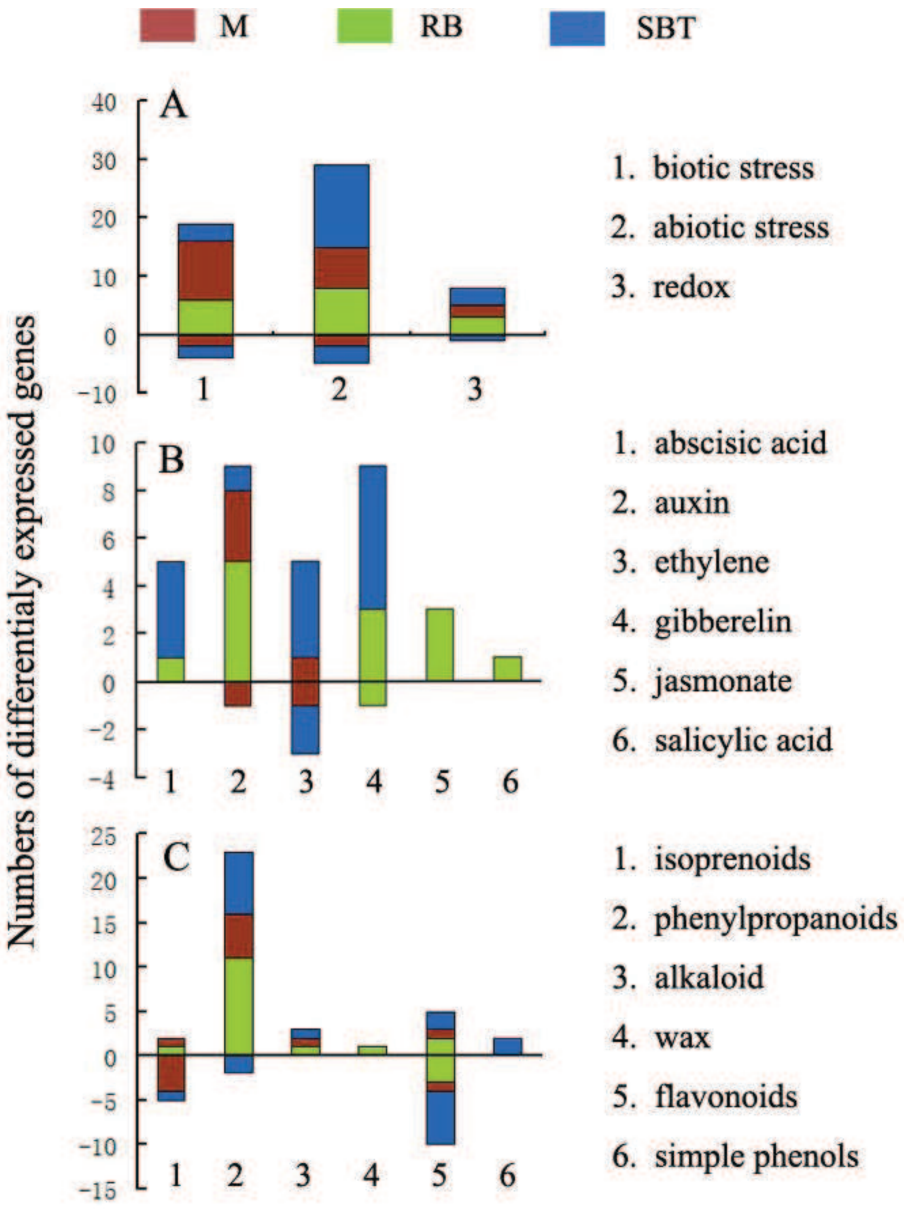

Additional File 5. Number of differentially expressed genes involved in stress and redox (A), hormone metabolism (B), and secondary metabolism (C). M, RB, and SBT represent Marsh grapefruit, Star Ruby grapefruit, and Sunburst mandarin, respectively. Positive axes represent the number of genes up-regulated, and negative axes represent the number of genes down-regulated.
