# Supplementary material for: Carotenoid accumulation affects redox status, starch metabolism, and flavonoid/anthocyanin accumulation in citrus
Source: BMC Plant Biol. 2015 Feb 3;15:27. doi: 10.1186/s12870-015-0426-4 (PMC4323224; doi:10.1186/s12870-015-0426-4)
Supplement: Additional file 6: — ABA contents in the ECMs and their wild-type controls. Columns and bars represent the means and ± SD, respectively (n = 3 replicate experiments). **indicates that the values are significantly different compared with wild type at the significance level of P < 0.01. M-33, RB-4, and SBT-6 represent the ECM lines of Marsh grapefruit, Star Ruby grapefruit, and Sunburst mandarin, respectively. [file 12870_2015_426_MOESM6_ESM.pdf]

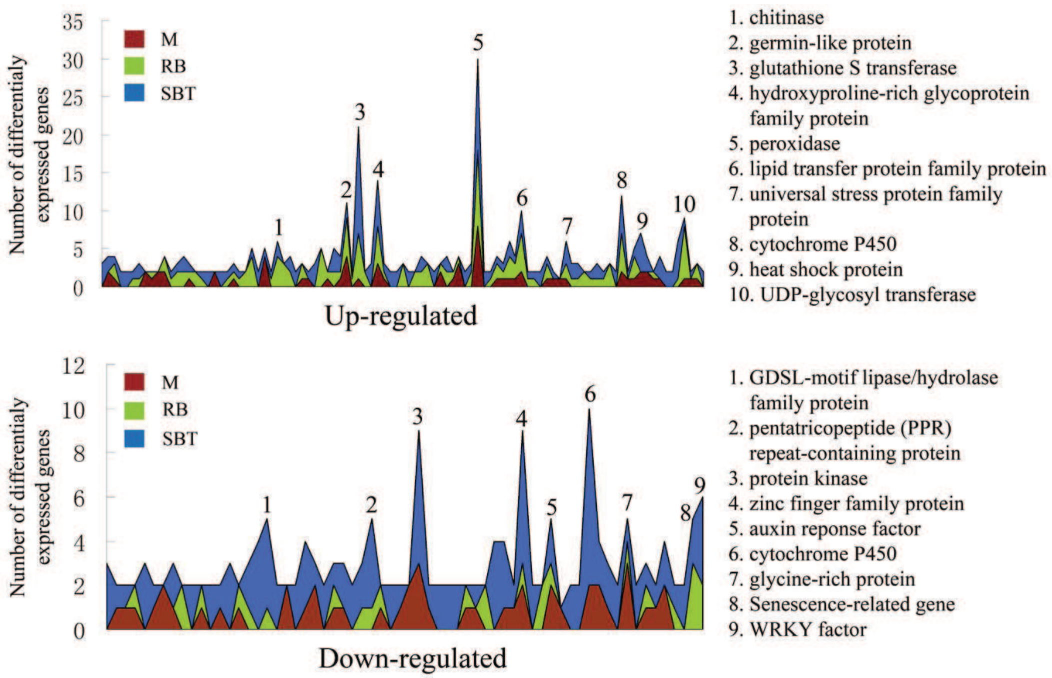

Additional File 6. Function annotation of predominantly detected genes. The graphs show number of genes annotated as the same function. The predominant functions are listed on the right of the graphs. M, RB, and SBT represent Marsh grapefruit, Star Ruby grapefruit, and Sunburst mandarin, respectively.
