# Supplementary material for: Carotenoid accumulation affects redox status, starch metabolism, and flavonoid/anthocyanin accumulation in citrus
Source: BMC Plant Biol. 2015 Feb 3;15:27. doi: 10.1186/s12870-015-0426-4 (PMC4323224; doi:10.1186/s12870-015-0426-4)
Supplement: Additional file 8: — Dry weight analysis of the wild types and ECMs. Columns and bars represent the means and ± SD, respectively (n = 3 biological replicate experiments. **indicates that the values are significantly different at the significance level of P < 0.01. [file 12870_2015_426_MOESM8_ESM.pdf]

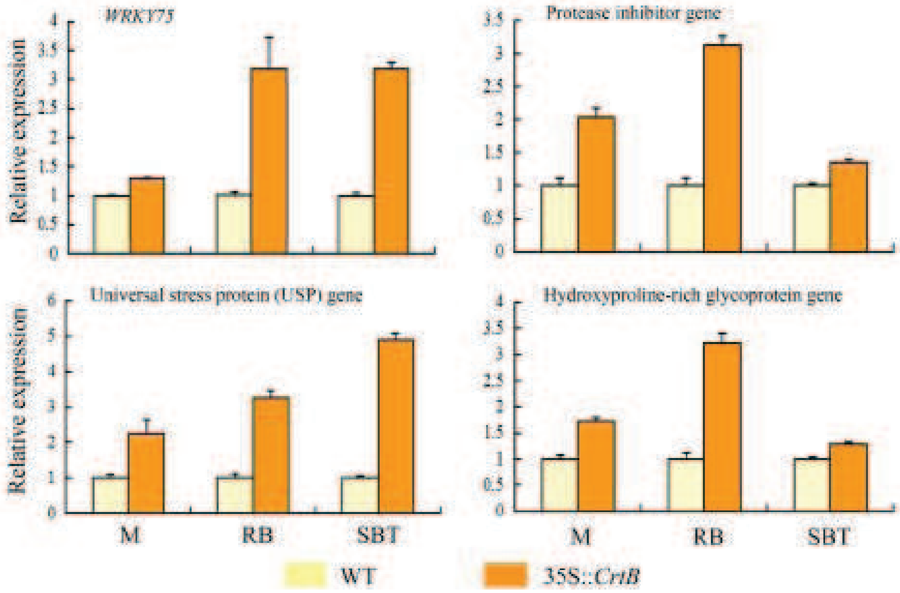

Additional File 8. Differentially expressed ROS-induced genes from the microarray data were verified in the calli via RT-PCR analysis. M, RB, and SBT represent Marsh grapefruit, Star Ruby grapefruit, and Sunburst mandarin, respectively. Trangenic calli (35S::*CrtB*) were the representative ECMs, M-33, RB-4, and SBT-6, which were also used for Affymetrix microarray analysis.
