# Supplementary material for: Carotenoid accumulation affects redox status, starch metabolism, and flavonoid/anthocyanin accumulation in citrus
Source: BMC Plant Biol. 2015 Feb 3;15:27. doi: 10.1186/s12870-015-0426-4 (PMC4323224; doi:10.1186/s12870-015-0426-4)
Supplement: Additional file 9: — Soluble sugar (fructose, glucose, and sucrose) contents in the ECMs and wild types. 35S:: CrtB represents the ECM lines. RB, M, HQC, and SBT represent Star Ruby grapefruit, Marsh grapefruit, Cara Cara navel orange, and Sunburst mandarin, respectively. Columns and bars represent the means and ± SD, respectively (n = 3 biological replicate experiments). * and ** indicate that the values are significantly different compared with wild type at the significance levels of P < 0.05 and P < 0.01, respectively. [file 12870_2015_426_MOESM9_ESM.pdf]

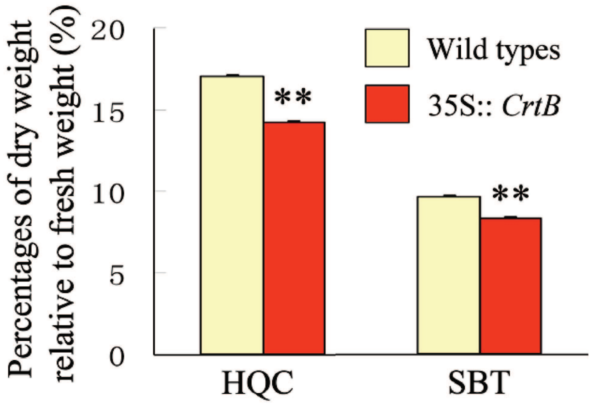

Additional File 9. Dry weight analysis of the wild types and ECMs (35S::*CrtB*). Columns and bars represent the means and ± SD, respectively (n = 3 biological replicate experiments. ** indicates that the values are significantly different at the significance level of $P < 0.01$.
