# Supplementary material for: Carotenoid accumulation affects redox status, starch metabolism, and flavonoid/anthocyanin accumulation in citrus
Source: BMC Plant Biol. 2015 Feb 3;15:27. doi: 10.1186/s12870-015-0426-4 (PMC4323224; doi:10.1186/s12870-015-0426-4)
Supplement: Additional file 10: — Cellular investigation and qRT-PCR analysis of the roots of Hongkong kumquats ( F. hindsii Swingle). (A) Cellular investigation of Hongkong kumquats. Ultrastructural inspection of dark-grown roots and embryoids. 35S:: CrtB represents the transgenic line. s, starch granules; p, plastoglobules; th, thylakoids; c, carotenoid crystal and characteristic internal membrane; ch, chromoplast; am, amyloplast. (B) qRT-PCR analysis of starch related genes in the roots of Hongkong kumquats. AMY, citrus sinensis alpha-amylase-like; SD1, α-amylase; SD2, α-amylase 3. (C) Expression levels of 6 stress-related and senescence-related genes that had been identified as differentially expressed between the ECMs and their wild types in microarray and qRT-PCR analyses. 1, WRKY75 (Cit.341.1.S1_s_at); 2, Protease inhibitor (Cit.16616.1.S1_at); 3, Universal stress protein (USP) family protein (Cit.14892.1.S1_at); 4, Hydroxyproline-rich glycoprotein family protein (Cit.37479.1.S1_at); 5, Senescence-related gene (Cit.14916.1.S1_at); 6, Plastocyanin-like domain-containing protein (Cit.5498.1.S1_at). (D) qRT-PCR analysis of key flavonoid biosynthetic genes in the roots of transgenic Hongkong kumquat and its control. CHS, chalcone synthase; CHI, chalcone isomerase. All transcript levels are expressed relative to WT (wild type). * and ** indicate that values are significantly different compared with wild type at the significance levels of P < 0.05 and P < 0.01, respectively. [file 12870_2015_426_MOESM10_ESM.pdf]

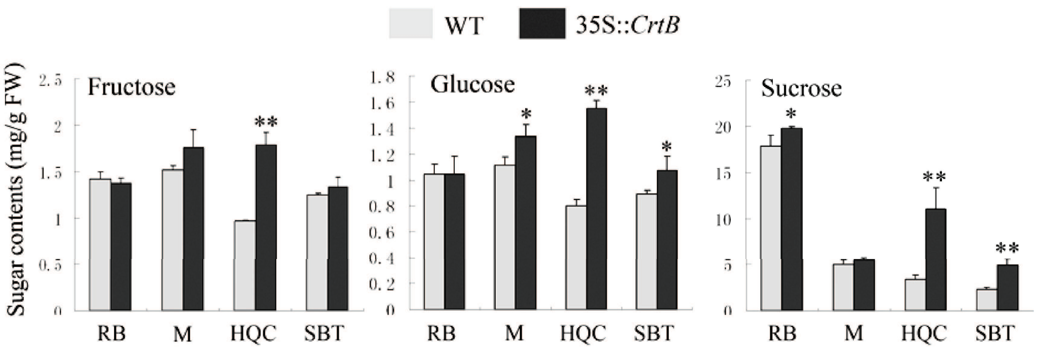

Additional File 10. Soluble sugar (fructose, glucose, and sucrose) contents in the ECMs and wild types. 35S:: *CrtB* represents the ECM lines. RB, M, HQC, and SBT represent Star Ruby grapefruit, Marsh grapefruit, Cara Cara navel orange, and Sunburst mandarin, respectively. Columns and bars represent the means and ± SD, respectively (n = 3 biological replicate experiments). * and ** indicate that the values are significantly different compared with wild type at the significance levels of $P < 0.05$ and $P < 0.01$, respectively.
