# Supplementary material for: Carotenoid accumulation affects redox status, starch metabolism, and flavonoid/anthocyanin accumulation in citrus
Source: BMC Plant Biol. 2015 Feb 3;15:27. doi: 10.1186/s12870-015-0426-4 (PMC4323224; doi:10.1186/s12870-015-0426-4)
Supplement: Additional file 12: — Specific primer pairs used for qRT-PCR analysis in the present study. [file 12870_2015_426_MOESM12_ESM.pdf]

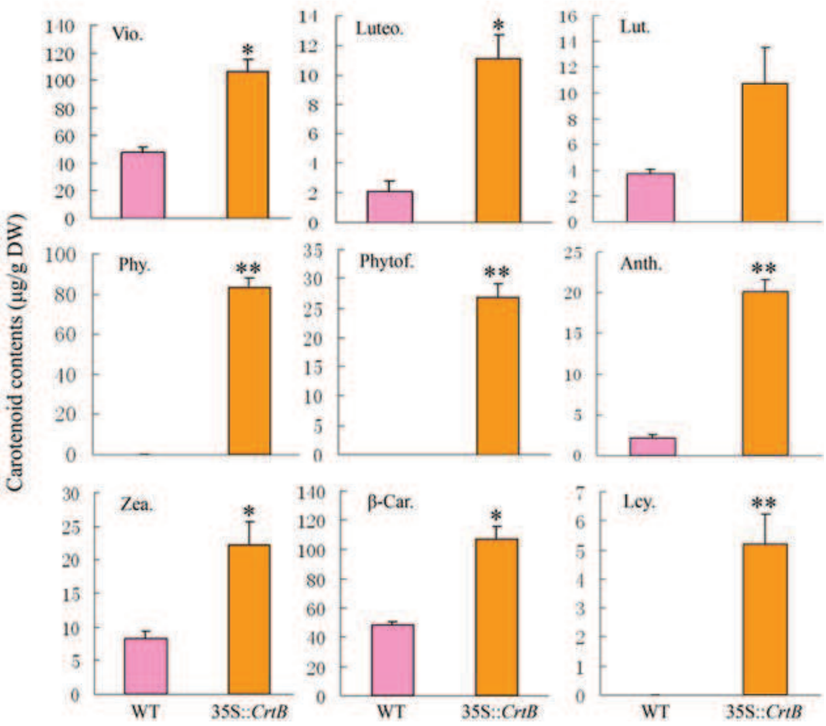

Additional File 12. Contents of various carotenoids in the *M. hupehensis* calli. Vio., Violaxanthin; Luteo., Luteoxanthin; Lut., lutein; Phy., Phytoene; Phytof., Phytofluene; Anth., Antheraxanthin; Zea., Zeaxanthin; β-Car., β-Carotene; Lcy., Lycopene. WT represents the light-cultured wild-type apple callus and 35S::*CrtB* represents the light-cultured transgenic apple callus with overexpression of *CrtB*. Columns and bars represent the means and ± SD, respectively (n = 2 replicate experiments). * and ** indicate that the values are significantly different compared with wild type at the significance levels of $P < 0.05$ and $P < 0.01$, respectively.

Reference:

Espley RV, Hellens RP, Putterill J, Stevenson DE, Kutty-Amma S, Allan AC: **Red colouration in apple fruit is due to the activity of the MYB transcription factor, MdMYB10.** *The Plant Journal* 2007, 49, 414-427.

Liao HL, Burns JK: **Gene expression in Citrus sinensis fruit tissues harvested from huanglongbing-infected trees: comparison with girdled fruit.** *Journal of*

Experimental Botany 2012, 63: 3307-3319.

Liu Q, Xu J, Liu YZ, Zhao XL, Deng XX, Guo LL, Gu JQ: **A novel bud mutation that confers abnormal patterns of lycopene accumulation in sweet orange fruit (*Citrus sinensis* L. Osbeck).** *Journal of Experimental Botany* 2007, 58: 4161–4171.

Liu Z, Ge XX, Wu XM, Kou SJ, Chai LJ, Guo WW: **Selection and validation of suitable reference genes for mRNA qRT-PCR analysis using somatic embryogenic cultures, floral and vegetative tissues in citrus.** *Plant Cell, Tissue and Organ Culture* 2013, 113: 469-481.
